# The Mechanism and Rate-Determining Step of Catalytic Ammonia Oxidation on Pd(332) at High Temperatures

**DOI:** 10.1021/acscatal.5c01448

**Published:** 2025-06-05

**Authors:** Jan Fingerhut, Jessalyn A. DeVine, Rongrong Yin, Mark E. Bernard, Alice Bremer, Dmitriy Borodin, Kai Golibrzuch, Theofanis N. Kitsopoulos, Daniel J. Auerbach, Hua Guo, Alec M. Wodtke

**Affiliations:** † Institute for Physical Chemistry, University of Göttingen, 37077 Göttingen, Germany; ‡ Max-Planck Institute for Multidisciplinary Sciences, 37077 Göttingen, Germany; § Department of Chemistry and Chemical Biology, Center for Computational Chemistry, 1104 University of New Mexico, Albuquerque, New Mexico 87131, United States; ∥ School of Mathematics and Natural Sciences, 5104 University of Southern Mississippi, Hattiesburg, Mississippi 39406, United States; ⊥ International Center for Advanced Studies of Energy Conversion, 37077 Göttingen, Germany

**Keywords:** NH_3_ oxidation, surface kinetics, intermediate detection, velocity-resolved kinetics, heterogeneous catalysis

## Abstract

Despite its immense practical importance in industrial production of nitric acid, the mechanisms of catalytic ammonia oxidation on platinum group metals remain controversial. In this work, we employ velocity-resolved kinetics to study ammonia oxidation on a model Pd(332) catalyst between 600 and 700 K. We obtain the temporal evolution of gas-phase reactants (NH_3_), products (NO, H_2_O) and—with the help of femtosecond laser-induced desorption—of a reaction intermediate, N*. The reaction exhibits the prompt appearance of H_2_O and the delayed formation of NO; the rate-determining step is the reaction N* + O* → N*O occurring at step sites. This means that N* is the longest-lived reaction intermediate, an insight that helps explain formation of byproducts like N_2_ and N_2_O. We present a mechanism that explains all experimental observations, based on transition-state theory calculations and using input from density functional theory. We also show that N*O desorption is accelerated by coadsorbed oxygen.

## Graphical abstract

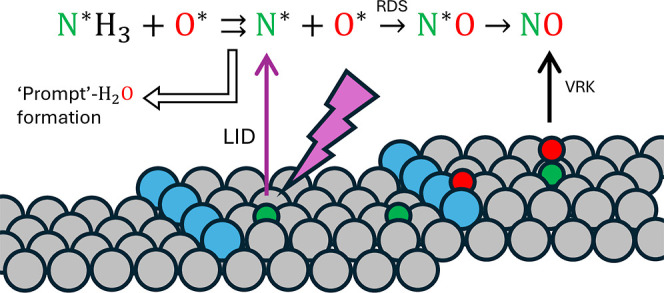

## 1 Introduction

Catalytic oxidation of ammonia (NH_3_) to nitric oxide (NO) and water (H_2_O) is the key step in the Ostwald process, which, together with the Haber–Bosch ammonia synthesis, accounts for most of the world’s production of nitric acid (HNO_3_) and mineral fertilizer.^1^ Catalytic oxidation of ammonia also produces the undesired side-product N_2_O, an important anthropogenic greenhouse gas.^2^ Modern catalytic processes must be sustainable; meaning, in case of the Ostwald process, that selectivity must be improved to reduce N_2_O emission. This is more likely to be achieved if the chemical reaction mechanisms involved in ammonia oxidation on platinum group metals can be confidently assigned. Unfortunately, neither mechanisms nor quantitative information on rates of elementary steps in heterogeneous catalysis are generally available^3^ and catalytic ammonia oxidation is no exception.

The NH_3_ oxidation reaction is typically carried out on platinum. In this work, we have studied ammonia oxidation on palladium (a member of the platinum group metals), whose reaction is thought to be similar to that on platinum.^4,5^ Palladium is itself an interesting catalyst as it is cheaper than Pt and used in alloy-nanoparticle synthesis to increase the selectivity of ammonia oxidation toward nitric oxide.^6,7^ Mechanisms have been proposed involving oxygen-assisted (eq 1) and hydroxyl-assisted (eq 2) hydrogen-loss

1 $$N^{*}H_{x}+O^{*}⇌N^{*}H_{x-1}+O^{*}H,\left(x=3,\;2,\;1\right)$$

2 $$N^{*}H_{x}+O^{*}H⇌N^{*}H_{x-1}+H_{2}O^{*},\left(x=3,\;2,\;1\right)$$

where * indicates the surface-coordinated atom. Subsequently, the products are formed by recombination (eqs 3 and 5) and disproportionation (eq 7).

3 $$N^{*}+O^{*}⇌N^{*}O$$

4 $$N^{*}O\to NO$$

5 $$N^{*}+N^{*}⇌N_{2}$$

6 $$N^{*}+N^{*}O⇌N_{2}^{*}O$$

7 $$O^{*}H+O^{*}H⇌O^{*}H_{2}+O^{*}$$

N_2_O is thought to be formed by recombination of N* with N*O (eq 6),^8,9^ suggesting a rational catalyst design approach based on finding reaction conditions that limit the concentrations of these two intermediates. Clearly, for this, determining the rate constants of the elementary reactions is of great importance in understanding catalytic selectivity in ammonia oxidation.

As ammonia oxidation is a complex sequential reaction, it is particularly important to identify the slowest reaction steps. These so-called rate-determining steps (RDSs) not only limit overall catalytic activity, but they also dictate which intermediates will exhibit the largest steady-state concentration. In ref 4, theoretical analysis found that N*O desorption (eq 4) is the RDS; but, since it is challenging to accurately model catalysis under realistic reaction conditions, experimental verification of this prediction would be useful. Unfortunately, neither the magnitudes of the rate constants of the elementary reactions nor the identities of important reaction intermediates have been determined experimentally.

In fact, experimental detection of catalytic reaction intermediates under reaction conditions is notoriously difficult. Time-resolved electron energy loss spectroscopy,^10^ reflection–absorption infrared spectroscopy,^11^ sum-frequency generation,^12^ secondary ion mass spectrometry^13^ and temperature-programmed X-ray photoelectron spectroscopy^14,15^ have all been used previously for this purpose. But these methods suffer from low sensitivity and/or poor time resolution—milliseconds to seconds.^16–20^ Even when sensitivity is sufficient, the poor time resolution often restricts the usefulness of these methods to temperatures far below realistic reaction conditions.

Velocity-resolved kinetics (VRK) in combination with microkinetic modeling has been shown to provide insights into active sites and active configurations in heterogeneous catalysis, revealing reaction mechanisms occurring at elevated temperatures.^21–23^ VRK has so far been restricted to measurements of the flux of thermally desorbing reactants and products, limiting its applicability in the analysis of complex sequential reactions. In this work, we overcome this limitation using laser-induced desorption (LID),^24^ where an ultrashort near-infrared laser pulse ejects N* intermediates from the surface during reaction. By detecting the ejected N atoms using resonance enhanced multiphoton ionization (REMPI), we directly obtain the time-dependent surface concentration of N* intermediate during ammonia oxidation on a palladium (332) single crystal surface between 603 and 703 K. These results—together with kinetic data on NH_3_, H_2_O and NO, and first-principles rate constant calculations—allow us to determine the reaction mechanism and the RDS for ammonia oxidation on palladium. We did not detect any other reactions products such as N_2_ and N_2_O because atomic oxygen is present in excess under the conditions of this work.^5^

The reaction exhibits prompt water formation with delayed NO production; furthermore, we show that the RDS is the reaction of N* with O* to form N*O occurring at undercoordinated Pd atoms found at step sites. The experimentally derived rate constant for this reaction compares favorably with TST predictions if the DFT predicted barrier height is increased by 0.17 eV. N*O desorption is faster than expected and not rate limiting due to repulsive interactions with adsorbed O* present under our reaction conditions. The kinetics revealed in this work show that long-lived and active nitrogen-atom adsorbates are an important characteristic of this catalytic system under high-temperature reaction conditions.

## 2 Results

### 2.1 Laser-Induced Desorption Velocity-Resolved Kinetics

Figure 1a shows kinetic traces measured at a surface temperature of 623 K for NH_3_ (×), H_2_O (□), and NO (—) when using a Pd(332) surface. Under similar reaction conditions using Pd(111), NO formation was not observed.^4,25,26^ The complete kinetic data with fits are provided in the Section S3. Desorption of unreacted NH_3_ and formation of H_2_O from Pd(332) occur within ∼300 μs, while NO is formed ∼100 times more slowly. Hence, we speak of prompt H_2_O formation and delayed NO formation. This two-time-scale mechanism indicates the importance of a relatively long-lived reaction intermediate, I*, corresponding to partially or fully dehydrogenated forms of ammonia (N*H_*x*_, where *x* = 2, 1 or 0), as shown by eq 8.

8 $${NH}_{3}\overset{F\left(t\right)}{\to }I^{*}\overset{k_{f}}{\to }N^{*}O\overset{k_{d}}{\to }NO$$

In eq 8, the delayed formation of NO from ammonia is modeled by assuming prompt formation of a reaction intermediate, I*, followed by N*O formation (*k*_f_) and N*O desorption (*k*_d_). Two solutions are obtained when one fits the kinetic trace of NO: a desorption-limited solution (*k*_d_ < *k*_f_, red ---) and an N*O formation limited solution (*k*_f_ < *k*_d_, blue —). Figure 1a shows fits to the NO kinetic trace; the two solutions give identical results. However, when the time-dependent surface concentration of N* is considered, only the NO formation limited case fits the data (Figure 1b). This simple analysis shows that desorption of N*O is not the RDS. Rather, the rate of formation of NO is limited by the reactivity of one of the N atom containing intermediates on the surface. We will return to this question after a brief aside into the LID detection of N*. We note here that under our experimental conditions, the kinetic data for N* always show a fast onset given by the temporal profile of the incident molecular beam and feature a single exponential shape. This observation is consistent with a single step toward N*O formation being rate-determining.

**Figure 1:**
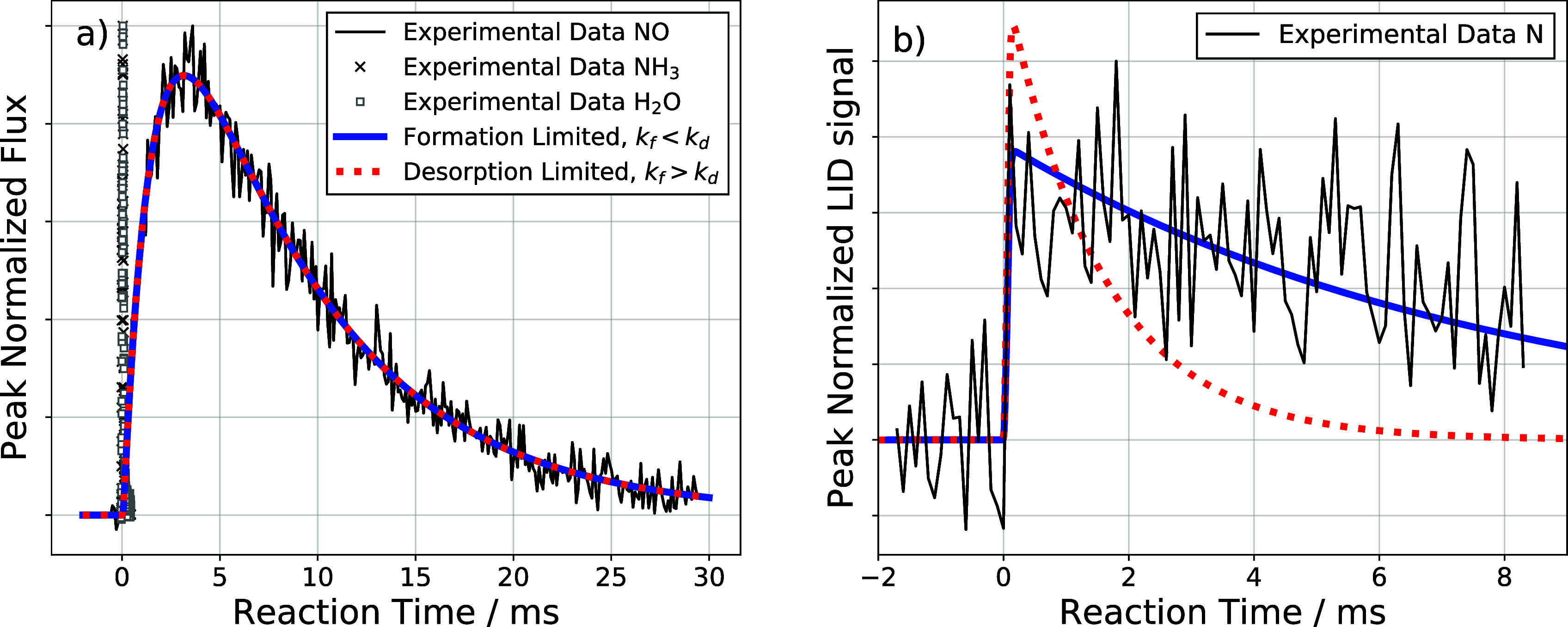
Kinetic traces of reactants, products and the N* intermediate. (a) Shows typical kinetic traces of gas phase reactants and products: NH_3_ (×), H_2_O (□) and NO (—) on Pd(332) at *T*_S_ = 623 K and a steady-state oxygen coverage of 0.15 ML. Also shown are fits to a formation limited model (*k*_f_ < *k*_d_, blue —) and a desorption limited (*k*_f_ > *k*_d_, red ---) model. (b) Shows the kinetic trace of N* under identical reaction conditions. Only the formation-limited model reproduces the experimental observations.

### 2.2 Laser-Induced Desorption of Reaction Intermediates

Our hope in using laser-induced desorption (LID) for reaction intermediate detection was that we would be able to eject adsorbates from the surface on a far shorter time scale than that of the oxidation reaction itself. This would allow the detected LID signal to reflect the status of the reaction at the time the desorption laser is fired without influencing the reaction rates. This was our impetus for using near-infrared femtosecond laser pulses, which directly excite substrate electrons, thereby inducing electronic temperatures on the order of several thousand Kelvin on a time scale of tens of femtoseconds. Increased phonon temperature subsequently arises due to electron–phonon coupling over tens of picoseconds. However, the peak phonon temperature is much lower than the electronic temperature due to the higher heat capacity of phonons relative to electrons.^27,28^ If LID proceeds dominantly by a hot electron-driven process, the desorption-inducing laser “plucks” the adsorbates from the surface on the order of 10^–13^ s.

Figure 2a shows that the N* LID signal indeed results from a hot electron-driven desorption process. Here, the delay between the desorption laser and the REMPI laser was varied to record the time required by the N atoms to fly from the surface to the ionization laser beam located ∼2 cm from the surface. The desorbed N atoms exhibit hyperthermal translational energies (Figure 2a inset); the effective translational temperature of the laser-desorbed N atoms (*T*_eff_ = 2779 K) is consistent with the high electron temperatures induced by the laser and is far larger than that of the surface (*T*_S_ = 473 K). In Figure 2b, we show a typical ion image of ejected N atoms at a given desorption-REMPI laser delay where we can clearly identify the LID signal by its hyperthermal speeds.

**Figure 2:**
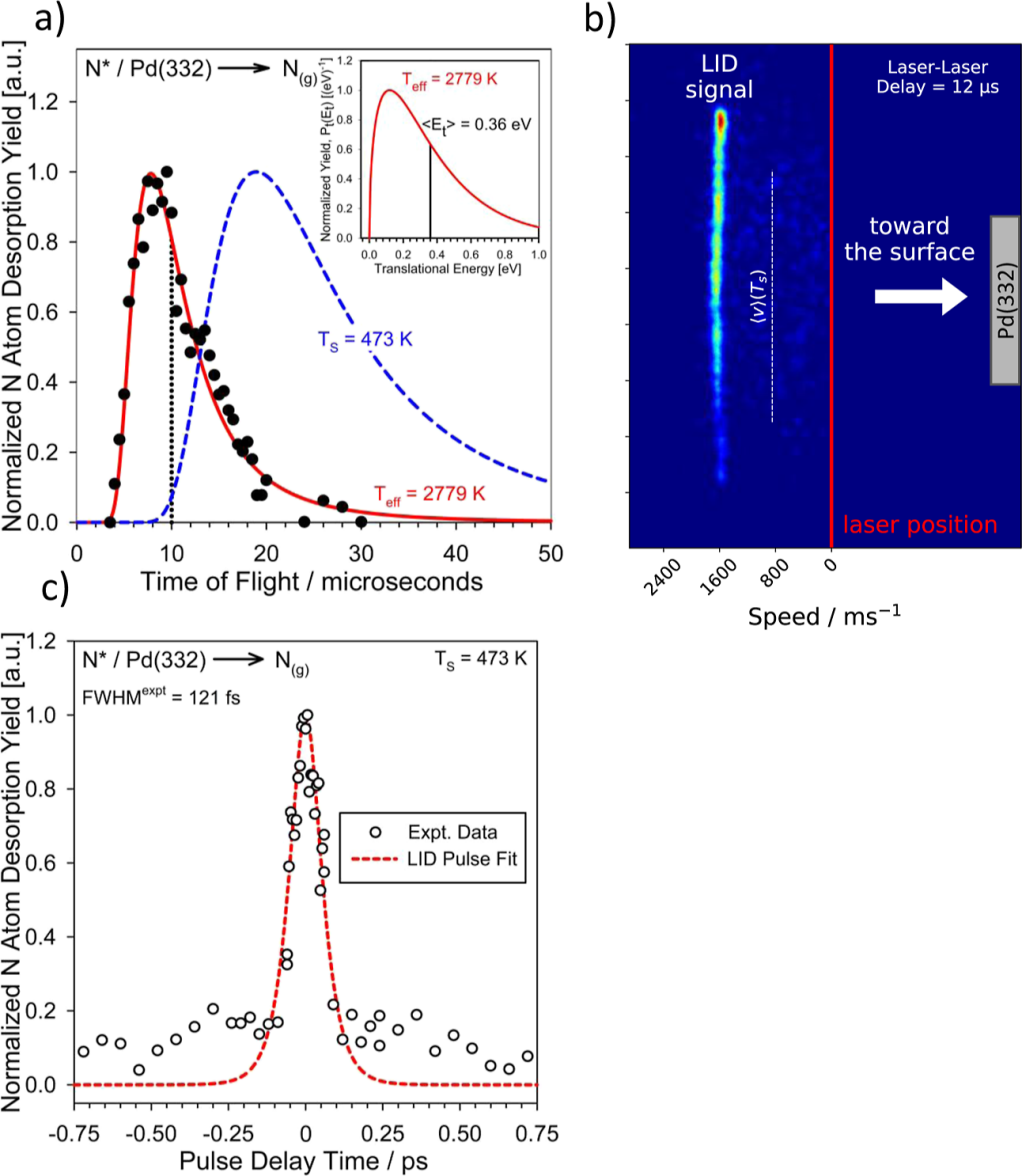
Laser-induced desorption of adsorbed N atoms. (a) Experimental time-of-flight distribution for N atom LID signal (●) determined by varying the time delay between desorption and ionization lasers at *T*_S_ = 473 K. Curves represent density-weighted Maxwell–Boltzmann distributions fit to experimental time-of-flight spectrum (red —; *T*_eff_ = 2779 K) & generated using the 473 K surface temperature (blue ---). N atom kinetic trace measurements discussed in this work were collected at a laser–laser delay corresponding to a time-of-flight of 10 μs (dotted black vertical line). The translational energy distribution for the LID N atom signal derived from converting the fitted Maxwell–Boltzmann distribution to the energy domain^31^ is displayed in the inset, where the solid black line reflects the calculated average translational energy for the desorbing N atoms of ∼0.36 eV. (b) A typical ion image for ejected N-atoms under reactions conditions for a given laser–laser delay of 12 μs is shown. The LID signal can clearly be distinguished by its hyperthermal speeds from thermal velocities. ⟨*v*⟩(*T*_s_) denotes the mean velocity of N atoms at 473 K. (c) Two-pulse correlation desorption yield of N atom intermediate (open points; fwhm ∼ 121 fs) as a function of pulse delay time on Pd(332) taken under ammonia oxidation conditions (*T*_S_ = 473 K) at a combined LID pulse energy of 400 ± 15 μJ. The dashed line represents the sech^2^ fit for the LID pulse duration (fwhm = 137 fs).

Further evidence for this conclusion is provided by Figure 2c showing the results of two-pulse correlation^29^ measurements. Here, the desorption laser pulse was split into two pulses of approximately equal energy (200 ± 15 μJ), with one pulse delayed with respect to the other (for experimental details, see Figure S6). The LID signal appears only when the two pulses closely overlap in time. Fitting the two-pulse correlation data (Figure 2c) to a form sech^2^(*t*/τ_p_), returns a value of τ_p_ = 68 fs. This corresponds to a full-width at half-maximum of 121 fs (the desorption-inducing laser was determined by autocorrelation to have a full-width at half-maximum of 137 fs). On this time-scale hot electrons have not yet relaxed to produce hot phonons.^29,30^

### 2.3 Identification of Active Site and Rate-Determining Step

In this section we first consider the dependence of the effective first-order rate constant *k*_f_^exp^ on oxygen coverage. This gives insights into the active site (Pd steps) involved in the RDS. We then present additional kinetic modeling and theoretical calculations that lead to the identification of the RDS being N* + O* → N*O.

Oxygen atoms on Pd(332) surfaces have three distinct binding sites at the up-step, down-step, and terrace.^32^ These binding sites are shown in Figure 3a. We used a kinetic model described in Section S2 of the Supporting Information to calculate the equilibrium distribution of oxygen atoms among these binding sites using DFT input (Figure 3b red, blue and black curves). Two functionals (PBE and RPBE) give nearly identical results. The populations of the three binding sites exhibit distinct dependence on mean oxygen coverage as they differ in number and stability. The most stable up-step site (—) fills preferentially at low mean coverage and becomes fully occupied while the population at the other two binding sites grows less rapidly. Scaled site-specific concentrations are compared in the same figure to the experimentally determined values of *k*_f_^exp^ (■). The derived rate constants closely follow the computed populations of O atoms at up-step sites, showing that the rate of formation of N*O is proportional to the concentration of O atoms at the up-step site.

$$k_{f}^{\exp}=k^{NH_{x}-O}\times \left[O_{us^{*}}\right]$$ 9

where $k^{NH_{x}-O}$ is the second-order rate constant for the RDS to form N*O. This conclusion is also consistent with the fact that we do not observe NO formation on Pd(111).

**Figure 3:**
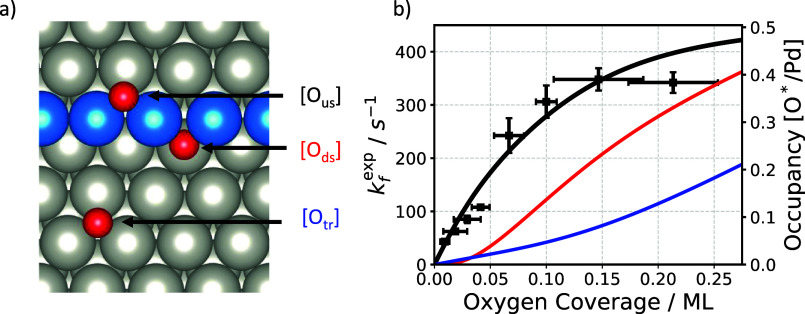
Identifying the active site of the rate-determining step. (a) The three types of binding sites for O atoms on Pd(332)—the up-step (us), down-step (ds) and terrace (tr). (b) The *k*_f_^exp^ results for *T* = 643 K are plotted versus the mean oxygen coverages as black squares. The black, red and blue lines show calculated equilibrium surface concentrations for up-step, down-step and terrace bound O atoms at *T* = 643 K which are obtained from parameters reported in previous work.^32^ Note that the equilibrium concentration of oxygen at the up-step is scaled to match the experimental rate constants best. We do not observe a better match with the experimental data by scaling the equilibrium concentration of oxygen at the down-step or the terrace. The same analysis is shown in the Section S3 and Figure S5 for other temperatures.

This insight suggests a kinetic mechanism with the RDS involving an oxygen atom bound at the up-step site that is responsible for sequential H-abstraction from NH_x_^*^ (*x* = 3, 2, 1) or N* recombination with O* to form N*O as formally written below.

10 $$N^{*}H_{3}+O_{us}^{*}\overset{k_{f,1}}{\to }N^{*}H_{2}+O^{*}H$$

11 $$N^{*}H_{2}+O_{us}^{*}\overset{k_{f,2}}{\to }N^{*}H+O^{*}H$$

12 $$N^{*}H+O_{us}^{*}\overset{k_{f,3}}{\to }N^{*}+O^{*}H$$

13 $$N^{*}+O_{us}^{*}\overset{k_{f,4}}{\to }N^{*}O$$

One of these four elementary processes must be the RDS; consequently, *k*_f_^exp^ corresponds to one of the following rate constants: *k*_f,1_[O_us_], *k*_f,2_[O_us_], *k*_f,3_[O_us_] or *k*_f,4_[O_us_]. Including NH_3_^*^ desorption, H_2_O formation by OH* disproportionation, and N*O desorption completes the mechanism. See Section S5 of the Supporting Information for more details.^33^

We next attempted to fit the kinetic traces of H_2_O and NH_3_ by setting one of these rate constants (*k*_f,*i*_,*i* = 1–4) equal to *k*_f_^exp^ and the others to a very high value (10^6^ s^–1^). Please note that the choice of RDS only influences the amplitude and not the shape of the N* kinetic trace and that the rate constants for OH–OH disproportionation were taken from prior work.^32^ The results of this analysis are shown in Figure 4 a–d, where we see predictions of the kinetic model for each choice of RDS. The fit is best for N* recombination with O* to form N*O as the RDS; however, the quality of fit is only slightly degraded for two other choices. DFT calculations (see Section S7) help resolve this ambiguity. Note that for our experimental conditions, the simulated water rates in Figure 4 c, d look very similar because for these choices of the RDS a large fraction of the H atoms initially bound to NH_3_ have been promptly converted to O*H. This dramatically accelerates the formation of H_2_O which is second order in the concentration of O*H.

**Figure 4:**
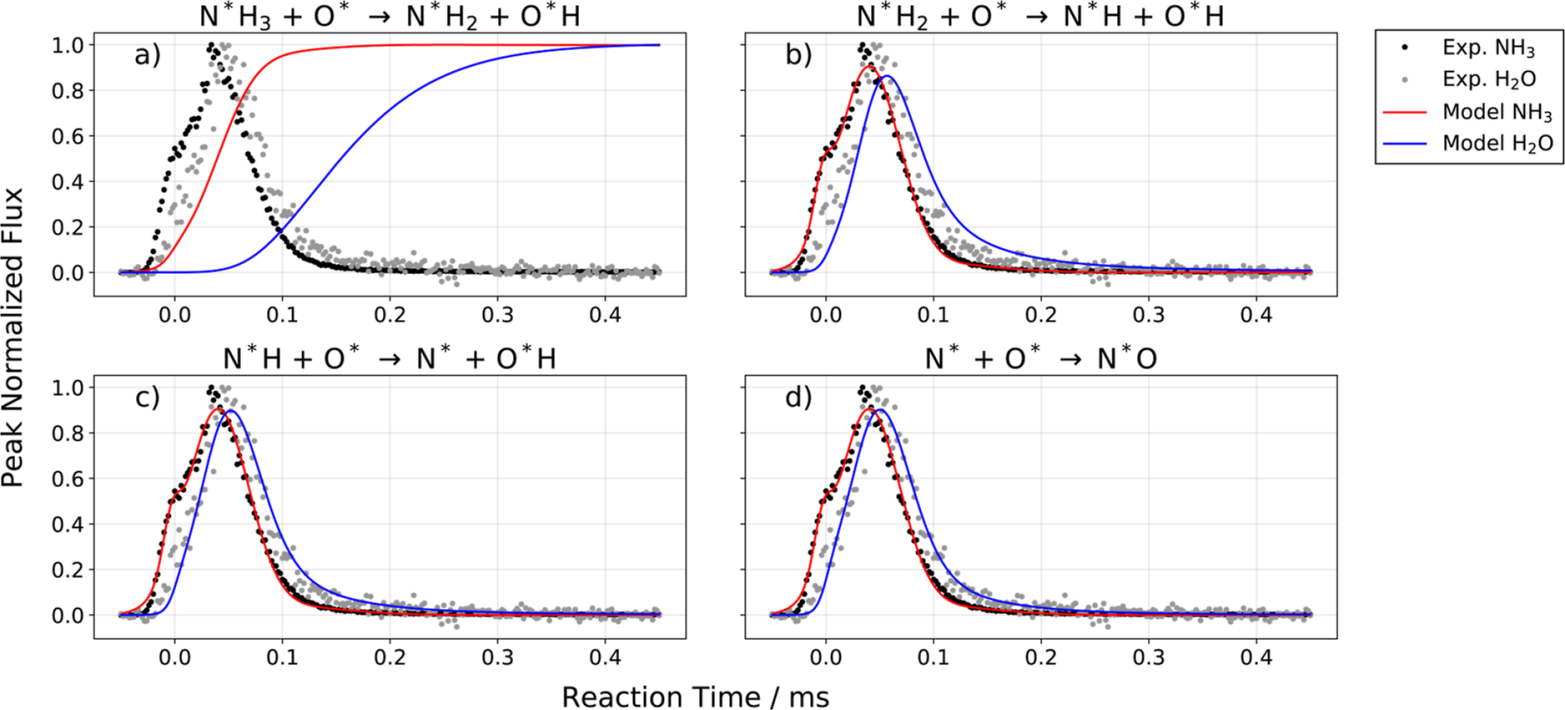
Consequence of RDS for water formation rate. Panels (a–d) show the experimental NH_3_ and H_2_O kinetic traces with the respective result of the kinetic model to the NH_3_ desorption (red solid line) and H_2_O formation (blue solid line) rate. The elementary step that is considered to be rate-determining is indicated above these panels. The experimental conditions are *T*_s_ = 623 K and [O]_ss_ = 0.009 ML. The low oxygen coverage leads to a low oxidation probability of 5%. In the proposed model, water formation is limited by the supply of O*H, thus, under these conditions we observe the biggest effect on the choice of the RDS.

Figure 5 shows a reaction path for the proposed mechanism involving reactions with O_us_^*^. Note that the barriers for reactions with O_us_^*^ are systematically lower than those with O_terr_^*^. The highest barrier along the path leading to formation of N*O is associated with recombination of N* with O_us_^*^ to form N*O (1.18 eV). Furthermore, the experimentally derived activation energy of the RDS (1.35 eV, see Figure 6 below) is consistent with this prediction. In light of the experimental and theoretical evidence, we conclude that the N* + O_us_^*^→ N*O reaction is the RDS.

**Figure 5:**
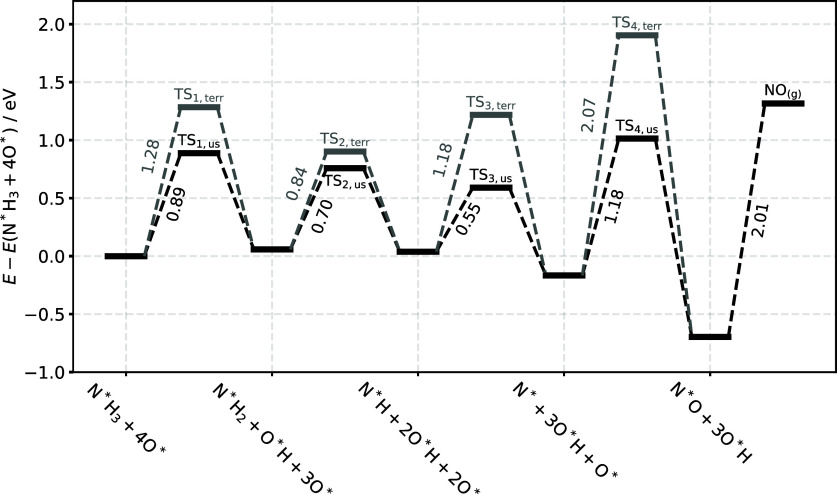
Energy diagram of O*-assisted ammonia oxidation on Pd(332) using RPBE. The black lines show the O_us_^*^—and the gray lines the O_terr_^*^—assisted pathway. The structures are provided in the Section S7 of Figures S12 and S13. The reference state for energy values is N*H_3_ and four O* in the infinite separation limit. The energies of the initial states are always given for the most favorable adsorption sites of the respective intermediate. Thus, the barrier height to TS_1,us_, TS_2,us,_ TS_3,us_ and TS_4,us_ include the step-terrace stabilization energies for NH_3_ (0.18 eV), NH_2_ (0.34 eV), NH (0.05 eV) and N (0.05 eV). The barrier height to TS_1,terr_, TS_2,terr_ and TS_3,terr_ include the step-terrace stabilization of O (0.11 eV). The barrier height of TS_4,terr_ includes the step-terrace stabilization of both O (0.11 eV) and N (0.05 eV). All values are given in eV and are not zero-point energy corrected.

**Figure 6:**
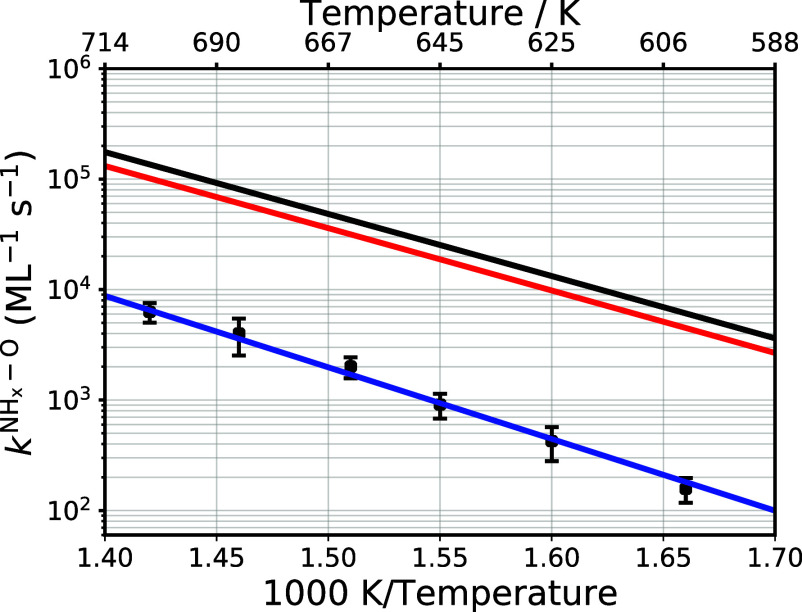
Arrhenius plot of experimental and theoretical second order rate constants of N*O formation. The black circles show the experimentally derived second-order rate constant for N*O formation. The Arrhenius parameters are *A* = 10^13.5±0.4^ (sML)^−1^ and *E*_a_ = 1.35 ± 0.05 eV. The red solid line shows the prediction of RPBE, the black solid line with PBE and the blue solid line the prediction of RPBE with a barrier adjustment of 0.17 eV.

We next computed the absolute rate constants for this reaction from transition-state theory (TST), see Section S7 and eqs S32 and S33. Figure 6 compares the experimentally derived rate constants to those obtained with TST using both the PBE (—) and RPBE (red —) functionals; the choice of functional alters the computed rate constants by only ∼30%.

The TST computed rate constants are about a factor of 10–20 larger than those derived from experiment (blue —), consistent with known uncertainties in DFT-derived barrier heights when using GGA functionals.^34^ We have earlier ruled out that desorption of N*O can be the RDS. In the next section we address this apparent contradiction to prior work.^4^

### 2.4 Effects of Oxygen Coverage on NO Desorption

Recall that the analysis described above provided effective formation rate constants *k*_f_^exp^—identified as *k*_*f*,4_[O_us_]—as well as N*O desorption rate constants, *k*_d_^exp^. Figure S15 summarizes the many measurements of *k*_d_^exp^ made in this work. The rate constants are, of course, strongly temperature dependent. Surprisingly, they are also strongly dependent on oxygen coverage. Figure 7 provides an overview of this behavior.

**Figure 7:**
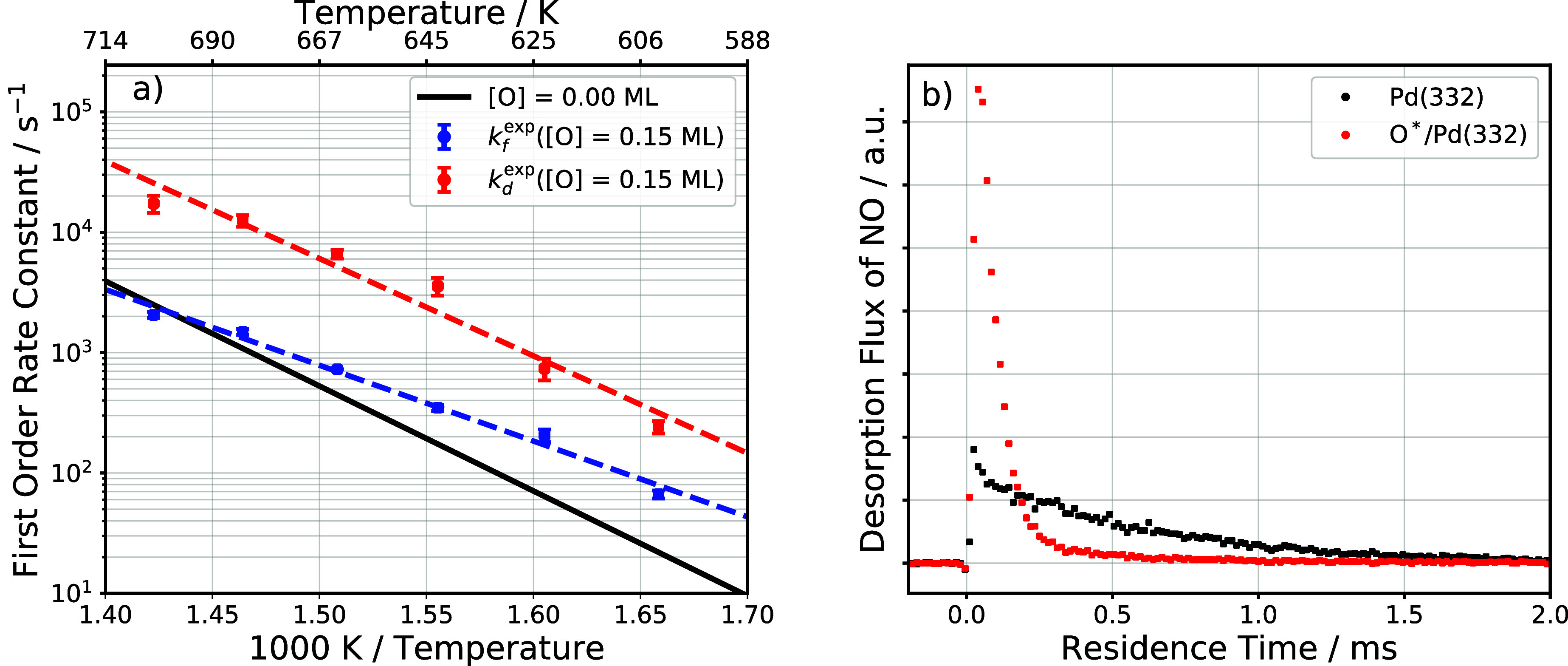
Influence of oxygen coverage on NO desorption rate. (a) Experimentally derived desorption rate constants of NO at zero oxygen coverage (black solid line),^35^ and 0.15 ML oxygen coverage (red circles). The blue circles show the effective first order formation rate constants of N*O at 0.15 ML oxygen coverage. The red and blue dashed line shows the Arrhenius fit to the respective rate constants. The general observation in our experiment is that the presence of coadsorbed oxygen increases the rate of NO desorption while the formation of NO is slower and shows a different temperature dependence. (b) Trapping desorption experiment for NO interacting with Pd(332) at two conditions: oxygen-free (•) and saturation coverage of oxygen (red •) on Pd(332). NO does not react with atomic oxygen; consequently, the integral of the kinetic traces are the same within 3%.

In Figure 7a, Arrhenius plots of *k*_d_^exp^ are shown for an O-coverage of 0.15 ML (red ---) and for zero O-coverage (—) reported previously.^35^ The derived activation energy decreases from 1.73 eV at zero oxygen coverage to 1.60 eV at 0.15 ML, indicative of a repulsive interaction between N*O and O*. The pre-exponential factor changes from 6.3 × 10^15^ s^–1^ at zero-coverage to 7.9 × 10^15^ s^–1^ at 0.15 ML, reflecting a modest reduction in N*O entropy with increasing O-coverage. While these Arrhenius parameters were derived from ammonia oxidation experiments, one can demonstrate this effect in a simpler experiment, where a beam of NO impinges upon a Pd(332) surface and desorption rates are measured from an oxygen-free and oxygen saturated surface, shown in Figure 7b. As expected from the considerations just given, desorption from an O-covered surface is much faster than that from a clean Pd(332) surface.

To find a microscopic interpretation of this effect, we turn to theory. DFT calculations using the RPBE functional show that N*O and O* compete for the same adsorption site; both O* and N*O adsorb most favorably at fcc-hollow-like up-step sites (Section S8b). Furthermore, O* can displace N*O, being bound more stably with respect to the second most favorable adsorption site (97 meV, versus 66 meV for N*O).^36,37^ DFT calculations also show (Figure S17) a repulsive interaction of at least 0.2 eV between O* and N*O when the two are placed at adjacent binding sites. This indicates that adsorption of even a single O* atom blocks multiple favorable adsorption sites for N*O thus lowering its entropy and that the energy penalty paid is similar to the change in activation energy seen above. This analysis provides a fundamental explanation of the empirical result that the desorption rate accelerates with O-coverage. It also helps us to understand why N*O desorption is not rate limiting in the ammonia oxidation reaction under our conditions.

## 3 Discussion

In this work, we have used VRK and LID to study NH_3_ oxidation on Pd(332) and derived a reaction mechanism and an associated kinetic model that describes the rate of formation of gas-phase NO, H_2_O as well as the desorption of NH_3_ and the kinetic behavior of the adsorbed N-atom intermediate. Analysis of the experimental data, combined with DFT calculations allowed us to identify that the rate-determining step is the recombination of N* with O* to form N*O. We furthermore observed a distinctive oxygen coverage dependence that indicates that the reaction occurs exclusively at step sites, consistent with observations that reactivity is absent on Pd(111).

We also computed this reaction’s second-order rate constants using DFT-TST. While DFT alone predicts values about one order of magnitude larger than those derived from experiment, an adjustment of +0.17 eV to the RPBE derived reaction barrier leads to quantitative agreement with experiment. This should not be considered an experimental determination of the reaction barrier. Errors in assumptions used to compute reactant and transition state entropies as well as transition state recrossing could also be important. We also note that adsorbate spin—neglected in this work—has been shown to contribute to the adsorbate entropy in recombinative desorption of H_2_ from Pt.^21^ How spin influences entropy in this reaction is unclear.

Previous theoretical work on ammonia oxidation on Pd(211) concluded that at temperatures of ∼800 K, NO desorption is the RDS.^4^ This is consistent with our calculations on the Pd(332) surface if we neglect the influence of oxygen-coverage on N*O desorption—compare the black and blue curves in Figure 7a. However, N*O desorption is strongly accelerated by coadsorbed oxygen atoms—compare the red and blue curves in Figure 7a. This can be understood using DFT calculations that reveal strong lateral interactions between N*O and O* as well as competition for the same adsorption sites. When ammonia oxidation is carried out in the presence of excess oxygen, it is unlikely that N*O desorption will be rate limiting.

The results of this work may also be useful in our thinking about NH_3_ oxidation on platinum.^4^ Indeed, there are distinct similarities between these two systems. The “prompt-H_2_O, delayed-NO” behavior seen for Pd in this work is consistent with temperature-programmed desorption measurements and electron energy loss spectroscopy to probe adsorbates for ammonia oxidation on platinum. That work showed N*H_*x*_ species present up to 380 K, whereas N* was present even up to 650 K.^38^ This suggests that N* is likely to be the most abundant reaction intermediate under conditions relevant to the Ostwald process carried out on Pt based catalysts. We reach the same conclusion for ammonia oxidation on Pd, as we found that N* conversion to N*O is the rate limiting reaction.

Our results also shed light on factors that influence catalytic selectivity in ammonia oxidation. Under the conditions of this work, we do not observe nitrogen-containing side products like N_2_ and N_2_O. While the mechanisms for formation of these two products in catalytic ammonia oxidation are not clearly understood, N_2_ formation presumably requires a high surface concentration of N atoms, while N_2_O formation requires both N* and N*O to be present at the surface. The results of this work show how increased oxygen coverage enhances NO formation, both by reducing the surface lifetime of N*O (thereby suppressing N_2_O formation) and by providing a pathway for N atom removal that competes effectively with N_2_ formation. This is consistent with suggestions from the literature that NO readsorption, which is not possible under our conditions, may be necessary for N_2_ and/or N_2_O formation.

## 4 Methods

### 4.1 Experimental Methods

The ammonia oxidation reaction was studied in a previously described^39^ custom ultrahigh vacuum apparatus using pulsed molecular beams of ammonia and molecular oxygen that intersected at a Pd surface with incidence angles of 30° and 0° with respect to the surface normal, respectively. Beam flux was calibrated as described in Section S1 of the Supporting Information. The beam of ammonia was produced by expansion of 10% ammonia in helium at 7 bar backing pressure through a piezo-actuated nozzle that produced a pulse of 35 μs duration with an incident translational energy of 0.16 eV. The oxygen beam was expanded similarly, either as a neat gas with 5 bar backing pressure (incidence energy 0.08 eV) or as a 5% mixture in 2.5 bar helium (incidence energy 0.30 eV). Gas-phase product kinetics were recorded in a pump–probe VRK experiment,^22^ where laser ionized products were produced 2 cm in front of the surface and detected on an ion imaging device as a function of delay between the ammonia and laser light pulses, while the oxygen beam was run asynchronously. The oxidation reaction is initialized by the molecular beam pulse of ammonia and the ratio of repetition rates (RRR) between the two molecular beams was used to control the steady-state oxygen coverage, which was determined using titration methods described in detail previously.^22,40^ We use eight steady-state oxygen coverages in this work ranging from $\frac{\left[O\right]}{{\left[O\right]}_{sat}}=0.028\;to\;0.640$ , where [O]_sat_ is the saturation coverage of 0.33 ML.^32^ The repetition rate of the NH_3_ molecular beam was set to 40, 20, 10, and 5 Hz based on the transient NO formation kinetics when they do not exceed 10, 50, 100, and 200 ms, respectively. NO formation rates are the slowest rates that we observed in this work. All experiments are conducted under isothermal conditions as indicated at surface temperatures varying between 603 and 703 K. We observe no memory effects on our acquired experimental data which would appear if experimental results depended on the order of execution. The outcome of all experimental results is determined by the known surface temperature and steady-state oxygen coverage. To account for the arrival time the incident NH_3(g)_ beam at the surface, we measured its time-profile and incident velocity in a separate experiment where the laser focus is moved to the position of the incident beam in the center of the ion optics and the molecular beam density as a function of the delay between the ionization laser and pulsed molecular beam is recorded.

The ion image was obtained using a pulsed homogeneous electric field (3 keV/cm) orthogonal to the scattering plane defined by the two molecular beams. This field accelerated ions to a time-gated chevron microchannel plate (MCP) ion/electron multiplier; the time-gate defined the ion’s mass-to-charge ratio. The MCP output was coupled to a phosphor screen, producing an illuminated spot at each point of impact which was recorded with a CCD camera. The in-scattering-plane velocities of ionized species obtained from the ion images were used to convert the product density to flux and to correct for the flight-time of the desorption products to the ionization laser.

Kinetics of adsorbed N atoms were recorded similarly as a function of time after the ammonia pulse using a pump–probe scheme, involving a desorption laser pulse at grazing incidence to the surface followed by an ionization laser pulse traveling parallel to the surface at a distance of 2 cm. For each molecular beam pulse of NH_3_ that arrives on the surface and initiates the reaction, a single molecular beam—ionization laser delay timing is chosen at which the LID signal is recorded. Subsequently, the system is allowed to relax back into its original configuration before the next molecular beam pulse arrives at the surface and the LID signal of the next delay timing is recorded. This guarantees that we measure the transient snapshot coverage of N*-atoms on the surface that is not influenced by the change of chemical composition due to the forced desorption of atomic/molecular species induced by the femtosecond laser pulse hitting the surface. Importantly, we note that we do not observe any measurable change in the reaction kinetics with and without the use of LID.

NH_3(g)_ and H_2_O_(g)_ were ionized by nonresonant multiphoton laser ionization using a Coherent Astrella Ti:sapphire laser (∼800 nm, pulse duration ∼ 137 fs, 300 μJ pulse energy). For detection of NO_(g)_, we used 1 + 1 resonance-enhanced multiphoton ionization (REMPI) through the X̃Π1/22→ÃΣ1/22 system (λ ∼ 226.25 nm). N* was desorbed with a <300 μJ laser pulse from the Ti:sapphire laser focused to a 1 mm spot on the surface and the desorbed N atoms were ionized by 2 + 1 REMPI exploiting the 2-photon 2s^2^2p^3^(^4^S_3/2_) →→ 2s^2^2p^2^3p­(^4^S_7/2_) resonance at 210.787 nm. Tunable light for REMPI was produced by a Nd:YAG-pumped dye laser system (Sirah Cobra stretch) equipped with nonlinear crystals for doubling and subsequent mixing (“3 x dye”). The Nd:YAG laser produced 532 nm light with 0.6 J per pulse at 10 Hz. We did not attempt to detect any other intermediate by using a different REMPI scheme.

A palladium single crystal (supplied by MaTeK) was polished to expose (111) and (332) facets.^21^ The sample was affixed to a 3-axis translation stage, which allowed the sample to move between these two facets and between the preparation and reaction chambers. At the start of each day of data acquisition, the surface was cleaned by sputtering with 10 μA of Argon ions (∼3 keV) for ca. 15 min and annealed at 923 K 15 min followed by further annealing at 1173 K for 3 min and the cleanliness confirmed by Auger electron spectroscopy.

### 4.2 Computational Methods

All periodic density functional theory (DFT) calculations were performed with the Vienna Ab initio Simulation Package (VASP),^41,42^ with spin polarization. Two different functionals, RPBE,^43^ and PBE^44^ within the generalized gradient approximation (GGA) were used to describe the exchange–correlation interaction. The electron–ion interactions were represented by the projector augmented wave (PAW) method.^45^

The Pd(332) surface was modeled by a five-layer slab, which has a (4 × 1) unit cell with the top two layers relaxed, separated by a 20 Å vacuum space to avoid interslab interaction. The Brillouin zone integration was performed on a 4 × 3 × 1 Monkhorst–Pack *k*-point mesh. The dipole correlation in the *z* direction was imposed to avoid the interaction between the vertically repeated images. The Kohn–Sham wave function of the valence electrons was expanded using plane waves with an energy cutoff of 400 eV. Fermi smearing with a width parameter of 0.1 eV was used. All geometries were optimized using a conjugate-gradient method until the forces acting on each atom were less than 0.02 eV/Å. The saddle points were determined using the climbing image nudged elastic band (CI-NEB) method^46^ and the dimer method,^47^ and confirmed by frequency calculations.

The adsorption energies were computed according to the following equation

14 $$E_{ads}=E_{adsorbate/slab}-\left(E_{adsorbate}+E_{slab}\right)$$

*E*_adsorbate/slab_, *E*_adsorbate_, *E*_slab_ are the energies of the adsorbed system, the gas-phase molecule, and the bare surface, respectively.

## Supplementary Material



## Notes

Molecular beam flux calibration, kinetic model for oxygen partitioning on Pd(332), kinetic modeling of NO and N*-data, pulse-pair correlation experiments, detailed kinetic model for NH_3_ oxidation, oxidation probability of NH_3_, TST-rate constant modeling of elementary steps in NH_3_ oxidation, influence of coadsorbed atomic oxygen on NO desorption (PDF)

## #

QuantaMap, 2333 CG Leiden, The Netherlands

## ¶

IBS Center for Quantum Nanoscience, Ewhayeodae-gil 52, Daehyeon-dong, Seodaemun-gu, 03760 Seoul, South Korea.
