# Supplementary material for: The Mechanism and Rate-Determining Step of Catalytic Ammonia Oxidation on Pd(332) at High Temperatures
Source: ACS Catal. 2025 Jun 5;15(12):10521–30. doi: 10.1021/acscatal.5c01448 (PMC12186266; doi:10.1021/acscatal.5c01448)
Supplement: Supplementary file 1 [file cs5c01448_si_001.pdf]

Supporting Information for: The Mechanism and Rate-Determining Step of Catalytic Ammonia Oxidation on Pd(332) at High Temperatures

Jan Fingerhut[1‡], Jessalyn A. DeVine[2‡†], Rongrong Yin[3], Mark E. Bernard[2], Alice Bremer[1], Dmitriy Borodin[1, 2‖], Kai Golibrzuch[2], Theofanis N. Kitsopoulos[2,4], Daniel J. Auerbach[2], Hua Guo[3] and Alec. M. Wodtke[1,2,5*]

‡These authors contributed equally to this work.

[1]Institute for Physical Chemistry, University of Göttingen, 37077 Göttingen, Germany.

[2]Max-Planck Institute for Multidisciplinary Sciences, 37077 Göttingen, Germany.

[3]Department of Chemistry and Chemical Biology, Center for Computational Chemistry, University of New Mexico, Albuquerque, New Mexico 87131, USA.

[4]School of Mathematics and Natural Sciences, University of Southern Mississippi, Hattiesburg, Mississippi 39406, USA.

[5]International Center for Advance Studies of Energy Conversion, 37077 Göttingen, Germany.

†Present address: QuantaMap, 2333 CG Leiden, The Netherlands.

‖Present address: Center for Quantum Nanoscience, Ewhayeodae-gil 52, Daehyeon-dong, Seodaemun-gu, Seoul 03760, South Korea.

*corresponding author: alec.wodtke@mpinat.mpg.de

Contents

S1. Molecular Beam Flux Calibration 3
S2. Oxygen binding on Pd(332): Diffusion and Site Partitioning 4
S3. Simplified kinetic modelling of NO and N* concentrations 6
S4. Pulse-pair-correlation experiments 11
S5. Detailed Kinetic Model of NH_3 Oxidation 12
S6. Oxidation Probability of NH_3 15
S7. TST modelling of the elementary steps in NH_3 oxidation 16
S8. Influence of oxygen coverage on NO desorption 23
a. Experimental desorption rate constants 23
b. DFT calculations 24
References 26

S1. Molecular Beam Flux Calibration

The calibration procedure used in this work is similar to a prior report[1], where the flux of molecules delivered by a molecular beam was determined from the increase in partial chamber pressure induced by the beam. The flux of incoming molecules can be calculated according to Eq. S1.

$$\mathrm{Flux} = \frac{\Delta pS}{Ak_bT_g} \qquad \text{Eq. S1}$$

In our work, Δp is the calibrated partial pressure of helium arising when a molecular beam of He enters the ultra-high vacuum chamber; S is the pumping speed of helium ($655\ \mathrm{L}s^{-1}$ according to the manufacturer[2]); A is the area of the molecular beam at the position of the surface and orthogonal to its propagation axis ($A = 3.1 \times 10^{-6}\ \mathrm{m}^2$); k_b is Boltzmann's constant and T_g is the temperature of the static gas in the chamber ($T_g = 296\ \mathrm{K}$). The molecular beam flux was calibrated using a pure He beam, and the partial pressure of He was measured with an off-axis quadrupole mass spectrometer (QMS) employing electron bombardment ionization. The detectors readings have been corrected by comparison to an ion gauge that was calibrated to $\mathrm{N_2}$ by the manufacturarer.[3] Using this corrected value of Δp, we find that the pure He beam operating at $10\ \mathrm{Hz}$ delivers 2.8×10^{13} molecules per cm^2 per pulse. We assume that the same total flux is achieved for the beam comprised of $10\ \%\ \mathrm{NH_3}$ in He, so that the flux of $\mathrm{NH_3}$ incident at the surface is 2.8×10^{12} molecules per cm^2 per pulse. We did not make corrections for the concentration of heavy species on the centerline of the supersonic molecular beam emerging from possible conductance limitations of pumping speed.[4]

S2. Oxygen Binding on Pd(332): Diffusion and Site Partitioning

O-atoms bind on Pd (332) at fcc hollows of terraces and can form zig-zag patterns at steps comprised of up-step O-atoms binding at a face-centered cubic (fcc) hollow site next to a monatomic step and down-step O atoms, binding at the fcc face of the (111) step.[5] The equilibrium population of O-atoms at these three binding sites is dependent upon temperature and mean oxygen coverage $[\mathrm{O}]$, which is the quantity measured in titration experiments[5-6] and is related to the site-specific coverages by Eq. S2.

$$[\mathrm{O}] = \frac{[\mathrm{O_{us}}] + [\mathrm{O_{ds}}] + 4 \times [\mathrm{O_{tr}}]}{6} \qquad \text{Eq. S2}$$

Here, us, ds and tr denote to binding at the up-step, down-step and terrace, respectively.

A kinetic model (Eq.'s S3-S13) was used to compute the equilibrium partitioning of oxygen atoms across these sites following previous work.[5]

$$\frac{\mathrm{d}[\mathrm{O_{us}}]}{\mathrm{d}t} = -k_{\mathrm{us}}^{\mathrm{tr}}[\mathrm{O_{us}}][\mathrm{tr}] + k_{\mathrm{tr}}^{\mathrm{us}}[\mathrm{O_{tr}}][\mathrm{us}] \qquad \text{Eq. S3}$$

$$\frac{\mathrm{d}[\mathrm{O_{ds}}]}{\mathrm{d}t} = -k_{\mathrm{ds}}^{\mathrm{tr}}[\mathrm{O_{ds}}][\mathrm{tr}] + k_{\mathrm{tr}}^{\mathrm{ds}}[\mathrm{O_{tr}}][\mathrm{ds}]\left(\frac{[\mathrm{O_{us}}]}{0.5\ \mathrm{ML}}\right) \qquad \text{Eq. S4}$$

$$\frac{\mathrm{d}[\mathrm{O_{tr}}]}{\mathrm{d}t} = -\left(\frac{\mathrm{d}[\mathrm{O_{us}}]}{dt} + \frac{d[\mathrm{O_{ds}}]}{dt}\right)\frac{1}{\mu^{-1} - 2} \qquad \text{Eq. S5}$$

$$\frac{\mathrm{d}[\mathrm{us}]}{\mathrm{d}t} = -2\frac{\mathrm{d}[\mathrm{O_{us}}]}{\mathrm{d}t} \qquad \text{Eq. S6}$$

$$\frac{\mathrm{d}[\mathrm{ds}]}{\mathrm{d}t} = -2\frac{\mathrm{d}[\mathrm{O_{ds}}]}{\mathrm{d}t} \qquad \text{Eq. S7}$$

$$\frac{\mathrm{d}[\mathrm{tr}]}{\mathrm{d}t} = -4\frac{\mathrm{d}[\mathrm{O_{tr}}]}{\mathrm{d}t} \qquad \text{Eq. S8}$$

where k_a^b represents the rate constant for hopping from one of the occupied binding sites of type a to an unoccupied binding site of type b. Here, $\mu = 1/6$ is the step density of Pd(332), and the concentration of surface site type a is $[a]$. We used the following initial conditions for the calculations.

$$[\mathrm{O_{us}}]_0 = [\mathrm{O_{ds}}]_0 = 0.5\ \mathrm{ML} \times \frac{[\mathrm{O}]}{[\mathrm{O}]_{\mathrm{sat}}} \qquad \text{Eq. S9}$$

$$[\mathrm{O_{tr}}]_0 = 0.25\ \mathrm{ML} \times \frac{[\mathrm{O}]}{[\mathrm{O}]_{\mathrm{sat}}} \qquad \text{Eq. S10}$$

$$[\mathrm{us}]_0 = 1 - 2[\mathrm{O_{us}}]_0 \qquad \text{Eq. S11}$$

$$[\mathrm{ds}]_0 = 1 - 2[\mathrm{O_{ds}}]_0 \qquad \text{Eq. S12}$$

$$[\mathrm{tr}]_0 = 1 - 4[\mathrm{O_{tr}}]_0 \qquad \text{Eq. S13}$$

Here $[\mathrm{O}]_{\mathrm{sat}} = \frac{1}{6}(0.5\ \mathrm{ML} + 0.5\ \mathrm{ML} + 4 \times 0.25\ \mathrm{ML}) = 0.33\ \mathrm{ML}$ is the saturation coverage of oxygen. These equations were parametrically solved to determine the steady state site-specific populations at $t = 1\,s$ as a function of mean oxygen coverage $[\mathrm{O}]$ and temperature. This time was chosen to guarantee that results are converged. Results are plotted in Figure S1 for the highest and lowest temperature of this work.

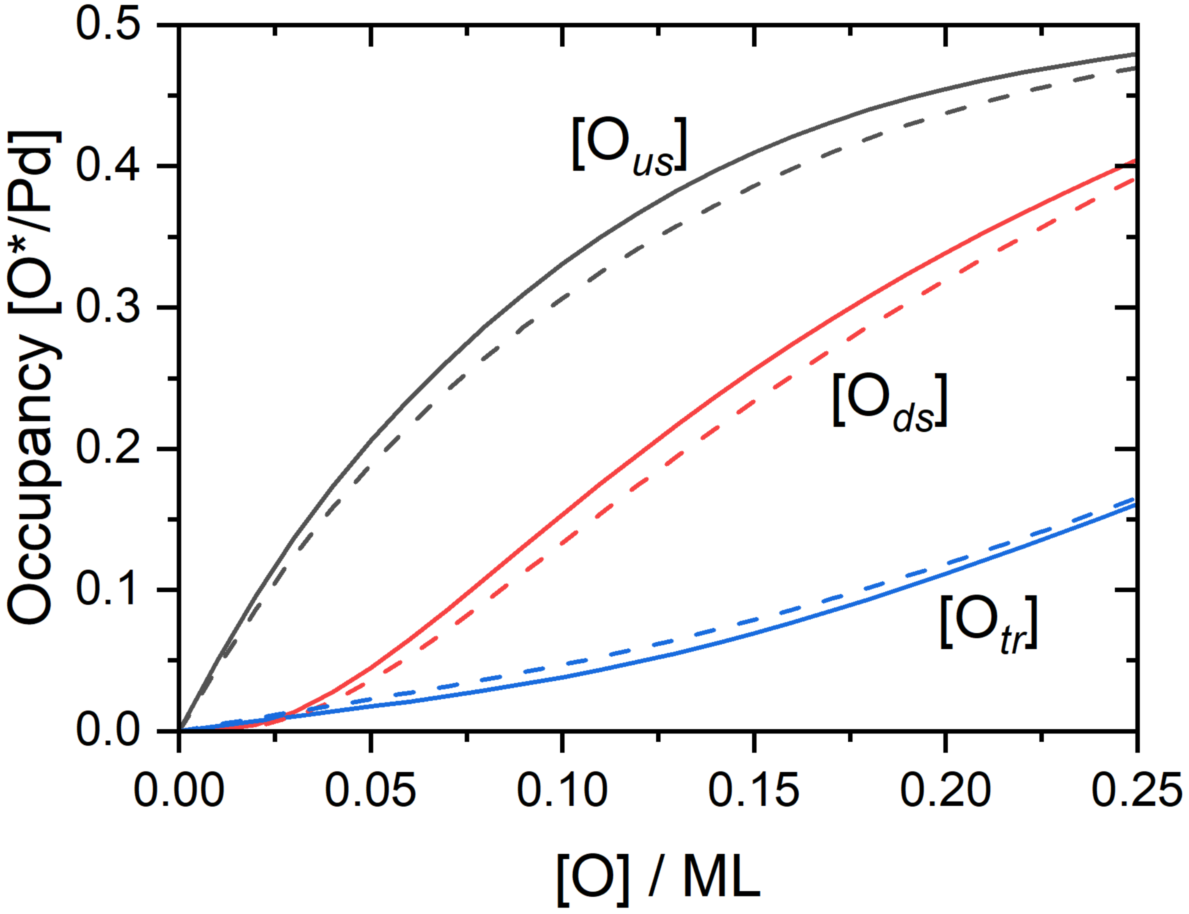

Figure S1: Equilibrium partitioning of oxygen atoms across three different binding sites of Pd(332) as a function of mean oxygen coverage*. The lines represent the evaluated differential equations at $603\ K$ (solid lines) and $703\ K$ (dashed lines). See text for further explanation of the figure.*

S3. Simplified kinetic modelling of NO and N* concentrations

We modelled the kinetics of $NO_{(g)}$ and N* assuming three reactions (Eq.'s S14-S16),

$$\mathrm{NH_3^g} \xrightarrow{F(t)} \mathrm{I^*} \quad \text{Eq. S14}$$

$$\mathrm{I^*} \xrightarrow{k_f^{\mathrm{exp}}} \mathrm{N^*O} \quad \text{Eq. S15}$$

$$\mathrm{N^*O} \xrightarrow{k_d^{\mathrm{exp}}} \mathrm{NO_{(g)}} \quad \text{Eq. S16}$$

governed by the following differential equations.

$$\frac{\mathrm{d[I^*]}}{\mathrm{dt}} = F^{\mathrm{NH_3}}(t) - k_f^{\mathrm{exp}}[\mathrm{I^*}] \quad \text{Eq. S17}$$

$$\frac{\mathrm{d[N^*O]}}{\mathrm{dt}} = k_f^{\mathrm{exp}}[\mathrm{I^*}] - k_d^{\mathrm{exp}}[\mathrm{N^*O}] \quad \text{Eq. S18}$$

$F^{\mathrm{NH_3}}(t)$ is the dosing function of $\mathrm{NH_3}$, which was obtained from a fit of the sum of three Gaussians to the experimentally obtained temporal profile of the $\mathrm{NH_3}$ beam. The ParametricNDSolve function in Mathematica was used to obtain a numerically defined functions for the time-dependent concentrations ($[\mathrm{X}](t; k_f^{\mathrm{exp}}, k_d^{\mathrm{exp}})$ for $\mathrm{X^*} = \mathrm{I^*}, \mathrm{N^*O}$), which depend parametrically on k_f^{exp} and k_d^{exp}.

The $NO_{(g)}$ data was fit to the functional form:

$$f_{\mathrm{NO}}(t; k_f^{\mathrm{exp}}, k_d^{\mathrm{exp}}, a, b) = a[\mathrm{NO}](t, k_f^{\mathrm{exp}}, k_d^{\mathrm{exp}}) + b \quad \text{Eq. S19}$$

to obtain the rate constants k_f^{exp} and k_d^{exp}. a is an amplitude and parameter and b accounts for the shift in the baseline. The resulting kinetic fits are shown in Figure S2 and Figure S3.

A similar expression was used to predict the adsorbed N-atom kinetic traces,

$$f_{\mathrm{N}}(t; \alpha) = \alpha[\mathrm{I^*}](t, k_f^{\mathrm{exp}}, k_d^{\mathrm{exp}}) + \beta \quad \text{Eq. S20}$$

where the rate constants were fixed to the values extracted from the NO kinetics measured under the same reaction conditions, and the vertical offset β was fixed to the experimental baseline. α is an amplitude parameter that is locally fitted to the respective kinetic trace.

The fitting procedure yields the results in Figure S4.

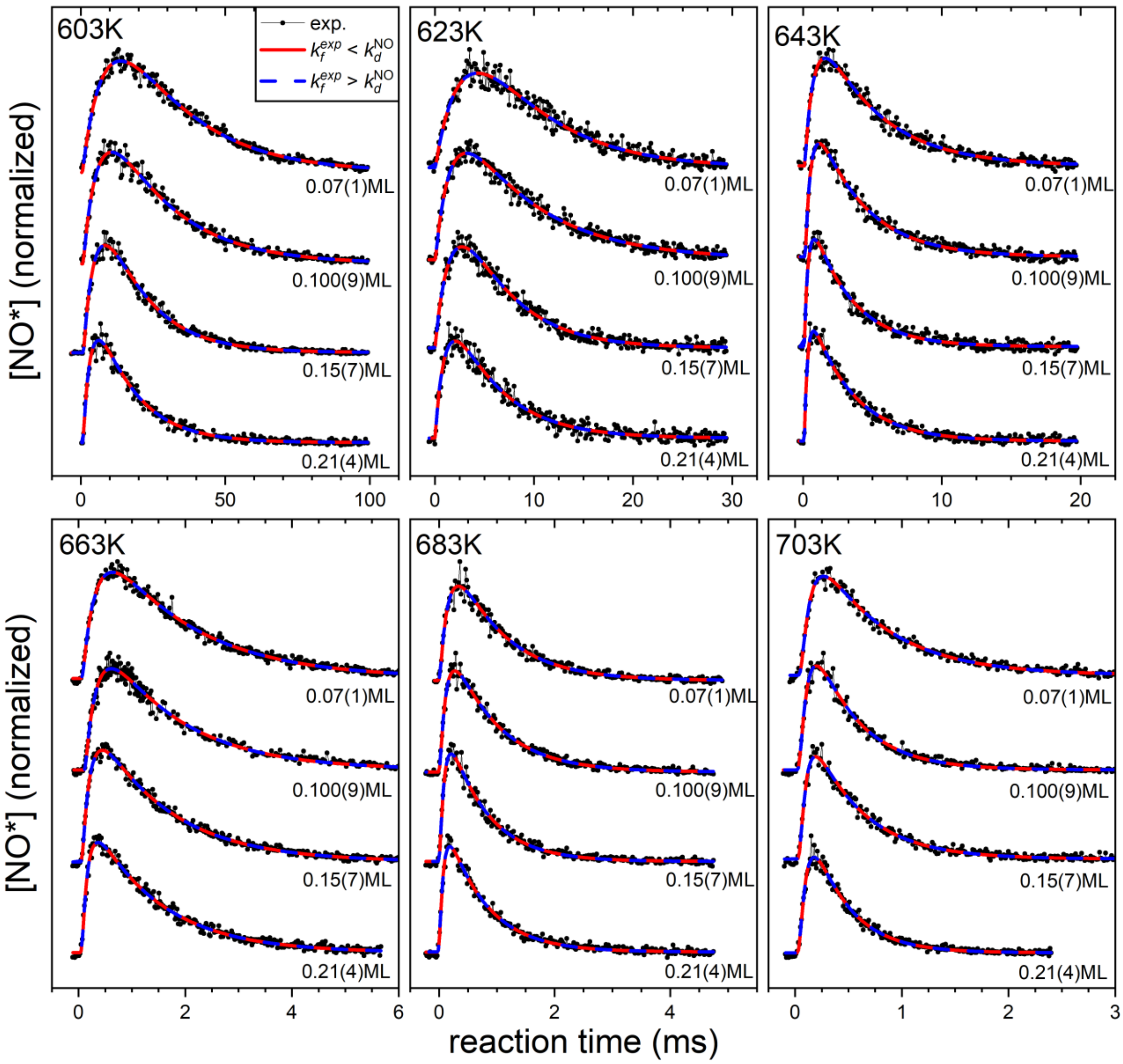

Figure S2: Fitting kinetics of NO formation to a simplified model of NH_3 oxidation on Pd(332). *This data set reflects moderation to high oxygen coverages. The red line reflects a mechanism where NO^* formation is rate limiting, while the blue line reflects a mechanism where NO^* desorption is rate limiting.*

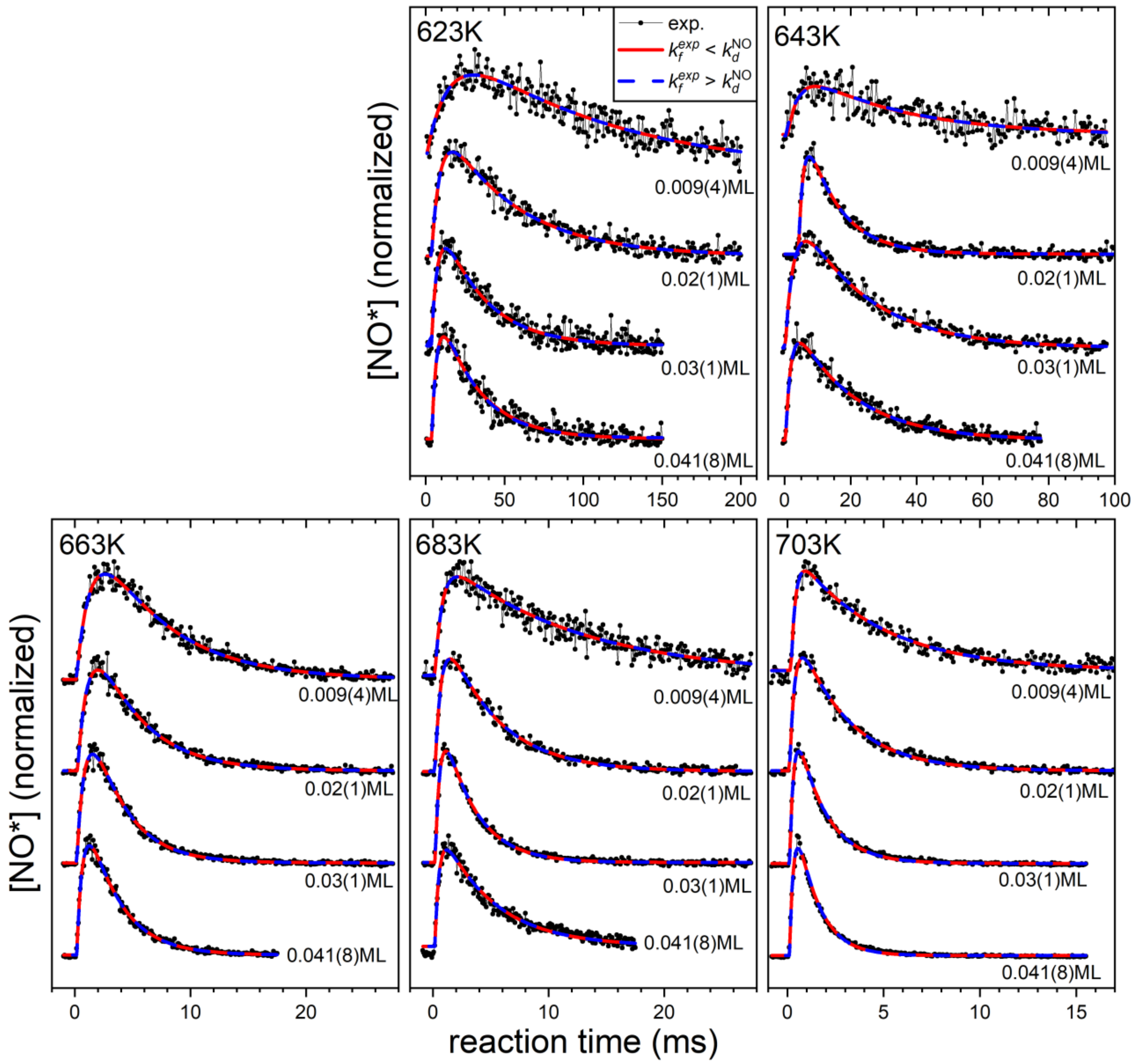

Figure S3: Fitting kinetics of NO formation to a simplified model of NH_3 oxidation on Pd(332). *This data set reflects low oxygen coverage. The red line reflects a mechanism where NO^* formation is rate limiting, while the blue line reflects a mechanism where NO^* desorption is rate limiting.*

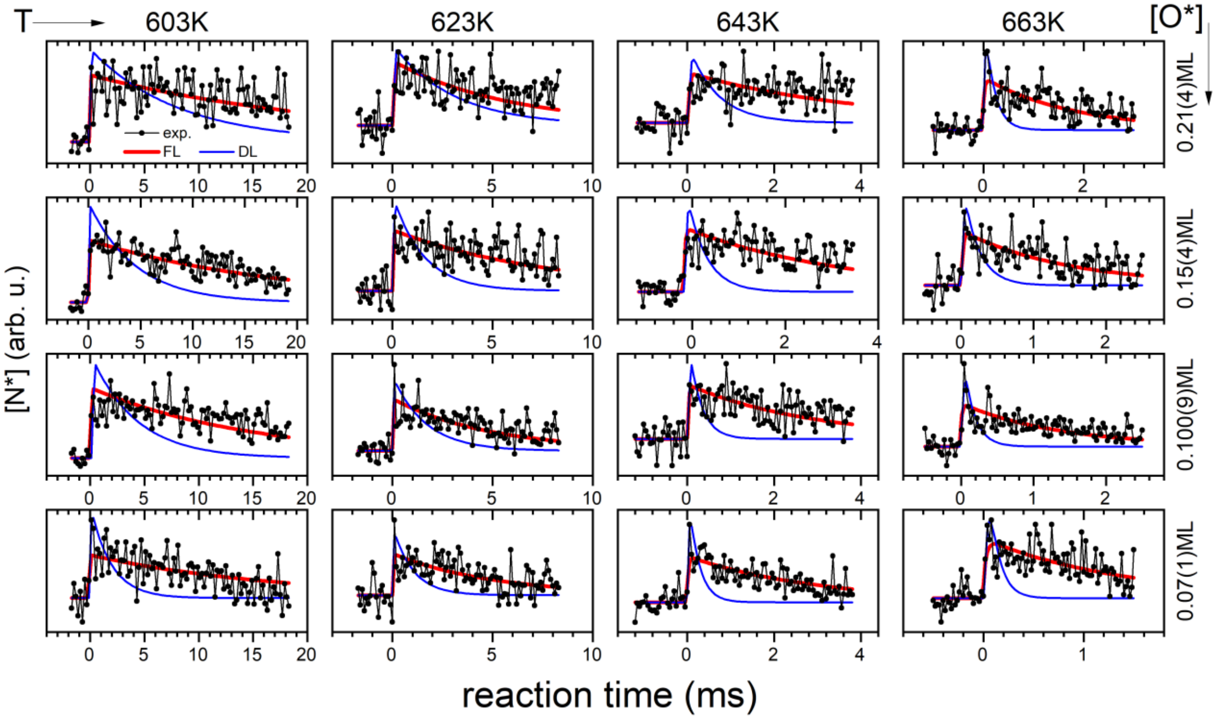

Figure S4: Fitting kinetics of N^* intermediate concentration to a simplified model of NH_3 oxidation on Pd(332). *The red line reflects a mechanism where NO^* formation is rate limiting, while the blue line reflects a mechanism where NO^* desorption is rate limiting.*

Figure S5 shows the derived dependence of k_f^{exp} on oxygen coverage as closed squares with error bars. This is compared to predictions of the kinetic model assuming exclusive reactivity of O-atoms at: us (black solid lines), ds (red solid lines) and tr (blue solid lines) sites.

$$k_f^{\mathrm{exp}} = k_s^{\mathrm{NH_x}} \times [\mathrm{O}_s] \qquad \text{Eq. S21}$$

where $s =$ us, ds or tr and $k_s^{\mathrm{NH_x}}$ is the second order rate constant of the rate-determining step. This analysis relies on the site-specific partitioning described in section S2. Best agreement is found assuming that up-step O atoms react exclusively with an N containing intermediate to form $\mathrm{N^*O}$.

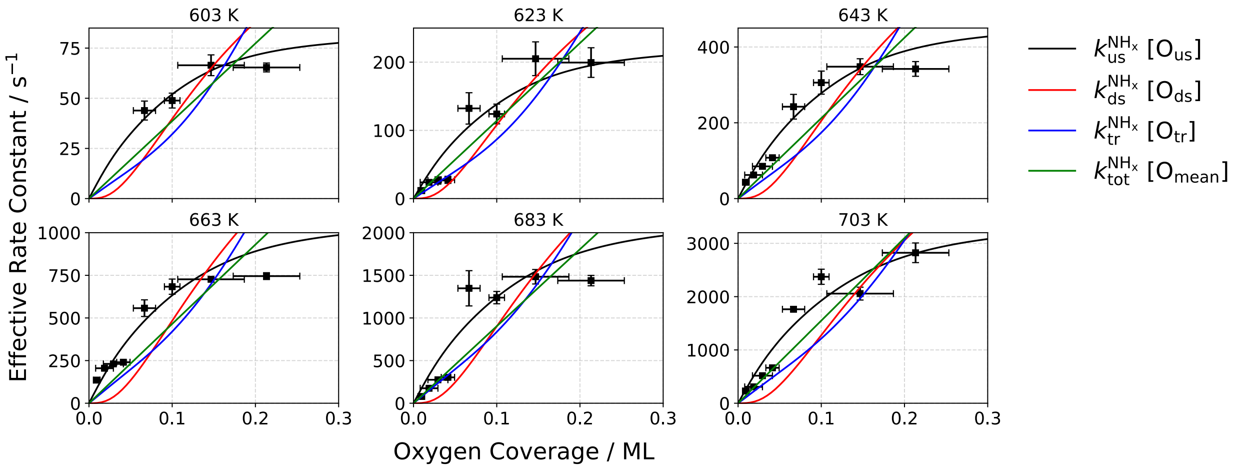

Figure S5: Evidence for reactivity of up-step Oxygen atoms. *Coverage dependence of* k_f^{exp} *(black squares) at the temperatures indicated, as well as fits to a single-reactive-site binding model, i. e.* $k_f^{exp} = k_s^{NH_x} \times [O_s]$ *for* s *= us, ds, and tr. The green solid lines show the scenario if all oxygen atoms react with the same probability.*

S4. Pulse-pair-correlation Experiments

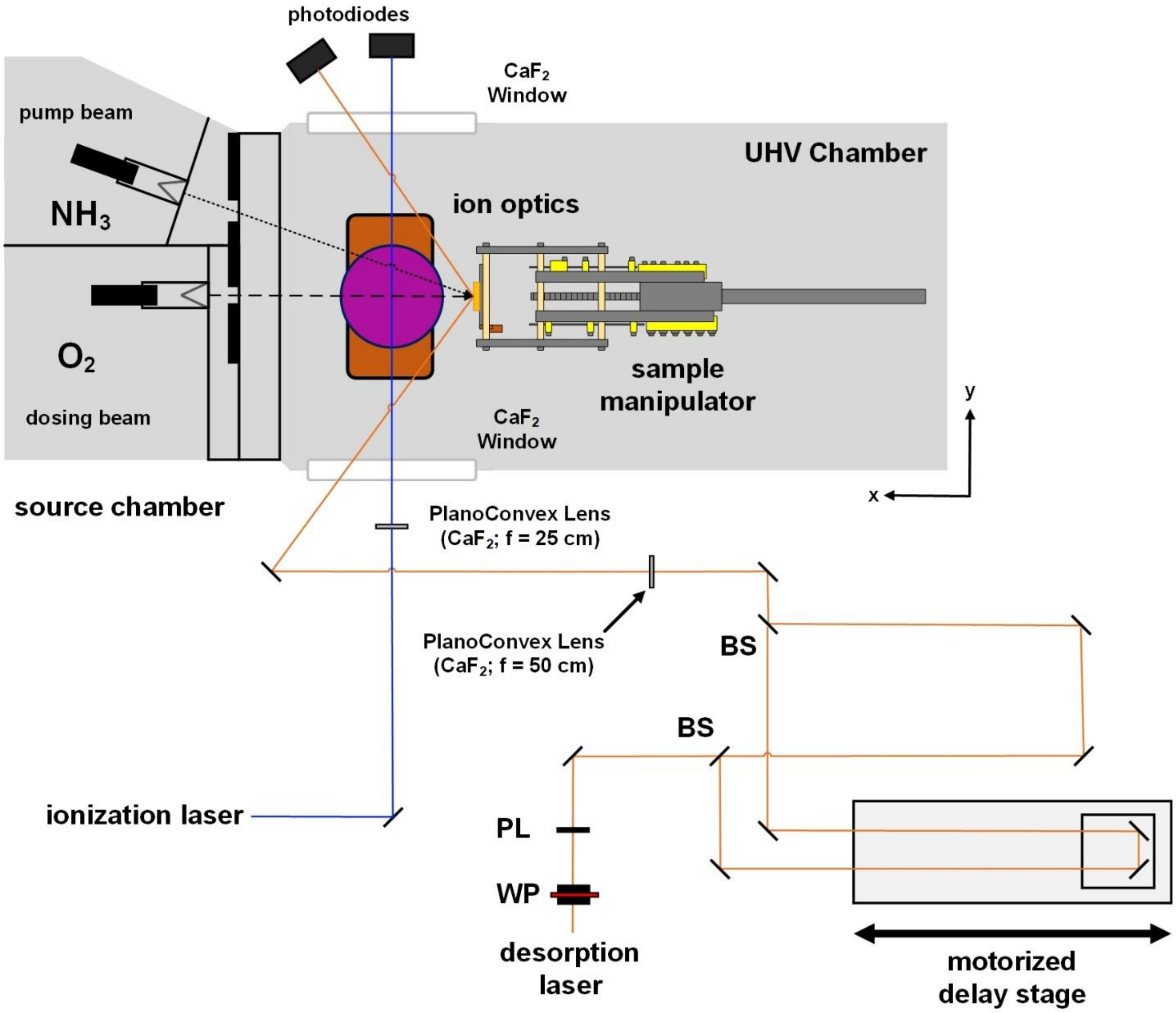

Figure S6: Schematic of the two-pulse correlation experiment. *A Ti:Sapphire (Coherent Astrella VF1K) desorption laser was used to deliver 800 nm pulses at a repetition rate of 10 Hz. The temporal pulse duration for the Ti:Sapphire (Coherent Astrella VF1K) laser pulses was determined using a Spectra-Physics pulseScout optical autocorrelator and found to be 137 fs. Pulse energies were controlled by a λ/2-plate (WP) and polarizer (PL). A beam splitter (BS) was introduced to split the laser light into two pulses, each of which contained roughly 50% of the total pulse energy measured prior to the splitter. The time delay between pulses was controlled using a 300 mm motorized delay stage (Thor Labs LTS300C) capable of 0.1 μm steps. The two beams were then spatially recombined to overlap at/on the Pd(332) surface. Nitrogen atom detection was accomplished through non-resonant multiphoton ionization using a LightConversion Carbide CB3-40W 100 kHz Ytterbium laser (1030 nm, 35 fs, 400 μJ/pulse) running at the same repetition rate as that of the molecular beam.* N*-atoms were formed on the Pd(332) surface by running the pulsed molecular beams of* NH_3 *in He and* O_2*.*

S5. Detailed Kinetic Model of NH_3 Oxidation

The differential equations associated with the kinetic reaction mechanism of $\mathrm{NH_3}$ oxidation are listed below.

$$\frac{\mathrm{d[NH_3]_{ad}}}{\mathrm{dt}} = F^{\mathrm{NH_3}}(t) - \left(k_d^{\mathrm{NH_3}} + k_f^{\mathrm{NH_2}}[\mathrm{O}]_{\mathrm{ad}}\right)[\mathrm{NH_3}]_{\mathrm{ad}} \quad \text{Eq. S22}$$

$$\frac{\mathrm{d[NH_2]_{ad}}}{\mathrm{dt}} = [\mathrm{O}]_{\mathrm{ad}}\left(k_f^{\mathrm{NH_2}}[\mathrm{NH_3}]_{\mathrm{ad}} - k_f^{\mathrm{NH}}[\mathrm{NH_2}]_{\mathrm{ad}}\right) \quad \text{Eq. S23}$$

$$\frac{\mathrm{d[NH]_{ad}}}{\mathrm{dt}} = [\mathrm{O}]_{\mathrm{ad}}\left(k_f^{\mathrm{NH}}[\mathrm{NH_2}]_{\mathrm{ad}} - k_f^{\mathrm{N}}[\mathrm{NH}]_{\mathrm{ad}}\right) \quad \text{Eq. S24}$$

$$\frac{\mathrm{d[N]_{ad}}}{\mathrm{dt}} = [\mathrm{O}]_{\mathrm{ad}}\left(k_f^{\mathrm{N}}[\mathrm{NH}]_{\mathrm{ad}} - k_f^{\mathrm{NO}}[\mathrm{N}]_{\mathrm{ad}}\right) \quad \text{Eq. S25}$$

$$\frac{\mathrm{d[NO]_{ad}}}{\mathrm{dt}} = k_f^{\mathrm{NO}}[\mathrm{O}]_{\mathrm{ad}}[\mathrm{N}]_{\mathrm{ad}} - k_d^{\mathrm{NO}}[\mathrm{NO}]_{\mathrm{ad}} \quad \text{Eq. S26}$$

$$\frac{\mathrm{d[OH]_{ad}}}{\mathrm{dt}} = [\mathrm{O}]_{\mathrm{ad}}\left(k_f^{\mathrm{NH_2}}[\mathrm{NH_3}]_{\mathrm{ad}} + k_f^{\mathrm{NH}}[\mathrm{NH_2}]_{\mathrm{ad}} + k_f^{\mathrm{N}}[\mathrm{NH}]_{\mathrm{ad}}\right) - 2k_f^{\mathrm{H_2O}}[\mathrm{OH}]_{\mathrm{ad}}^2 \quad \text{Eq. S27}$$

$$\frac{\mathrm{d[H_2O]}}{\mathrm{d}t} = k_f^{\mathrm{H_2O}}[\mathrm{OH}]_{\mathrm{ad}}^2 \quad \text{Eq. S28}$$

$F^{\mathrm{NH_3}}(t)$ is $\mathrm{NH_3}$, dosing function defined above in section S3. The integral of the function was normalized to the calibrated beam flux (2×10^{-3} ML per pulse) as described in section S1. $k_d^{\mathrm{NH_3}}$ and k_d^{NO} are the desorption rate constants of $\mathrm{NH_3}$ and NO and $k_f^{\mathrm{H_2O}}$ is the $\mathrm{OH-OH}$ disproportionation rate constant determined previously.[5] k_d^{NO} was taken from the simplified kinetic analysis presented in section S3. $k_d^{\mathrm{NH_3}}$ was set arbitrarily to $10^6\ \mathrm{s^{-1}}$, making the time scale of ammonia desorption much faster than the experimental temporal resolution. The ratio of $k_d^{\mathrm{NH_3}}$ and $k_f^{\mathrm{NH_2}}[\mathrm{O}]_{\mathrm{ad}}$ was chosen to match the experimentally determined oxidation probability of $\mathrm{NH_3}$, presented in section S6. (Note that this must be treated differently when we consider the possibility that $\mathrm{N^*H_2}$ formation is rate limiting, in which case $k_d^{\mathrm{NH_3}}$ is set to zero.) The different scenarios discussed in section 2 of the main text are computed by assuming that all but one reaction of $\mathrm{NH_x}$ (x = 3, 2, 1, 0) with oxygen are fast ($k_f^{\mathrm{NH_x}}$ is set to $10^6\ \mathrm{s^{-1}}$). For example, if the rate-determining step is assumed to be $\mathrm{N^*O}$ formation, $k_f^{\mathrm{NO}}[\mathrm{O}]_{\mathrm{ad}}$ is set equal to k_f^{exp} and obtained from the fit to the NO data, while the other rate constants are set to $10^6\mathrm{s^{-1}}$.

Note that in this analysis, only the amplitude and not the shape of the $\mathrm{N^*}$-kinetic trace depends on the choice of the rate limiting step. Furthermore, our mechanism neglects reactions with the $\mathrm{O^*H}$-species, an assumption justified by the experimental conditions. Specifically, under all our experimental conditions a steady-state coverage of oxygen of

at least 10^{-2} ML is prepared. The exposure of NH_3 is one order of magnitude lower and at the same time the reaction probability was at maximum 25%, see section S6. Hence, there was initially much more O^* than OH^*, which favors reactions between N^*H_x and O^* over reactions with O^*H.

We also note that the rates of NO formation seen in this work always exhibit a single exponential decay indicative of pseudo-first order kinetics. This is strong evidence that the RDS involves atomic oxygen as this is the only species able to exhibit steady-state behavior over the course of the reaction. While we cannot rule out that fast reactions with O^*H are involved in the fast water formation, this does not contradict our findings about the RDS and hence the main conclusions of this work.

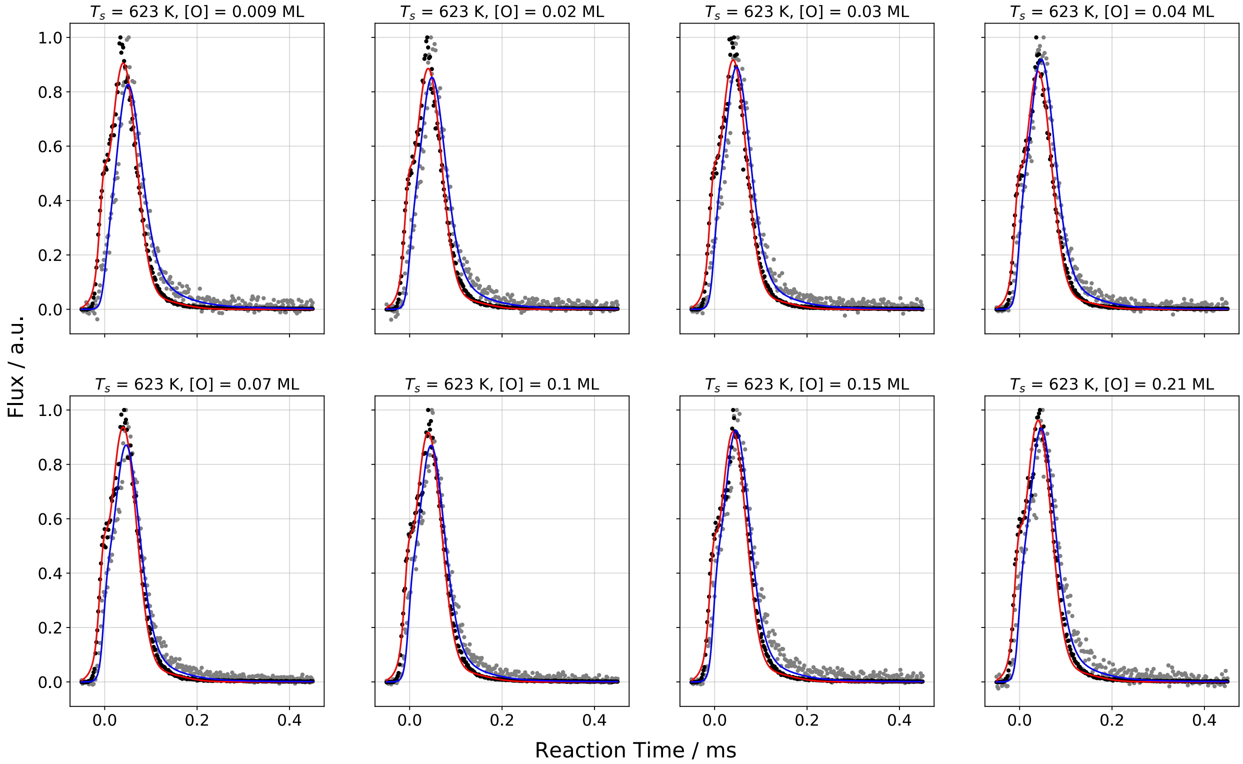

Figure S7: Kinetic Traces for NH_3 (●) and H_2O (●) at a reaction temperature of 623 K are consistent with N^*O formation being the RDS. *The red and blue solid line show the result of the derived kinetic model if N^*O formation is assumed to be the RDS.*

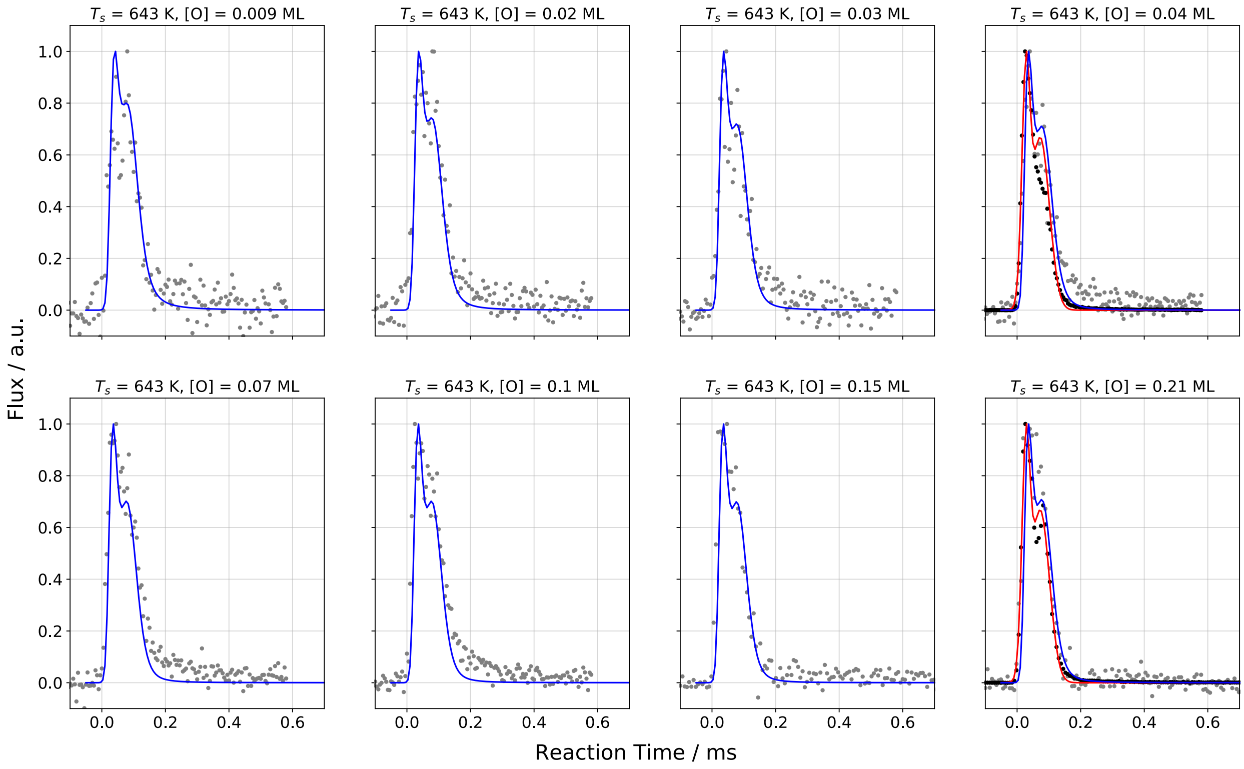

Figure S8: Same as Figure S7 but at 643 K.

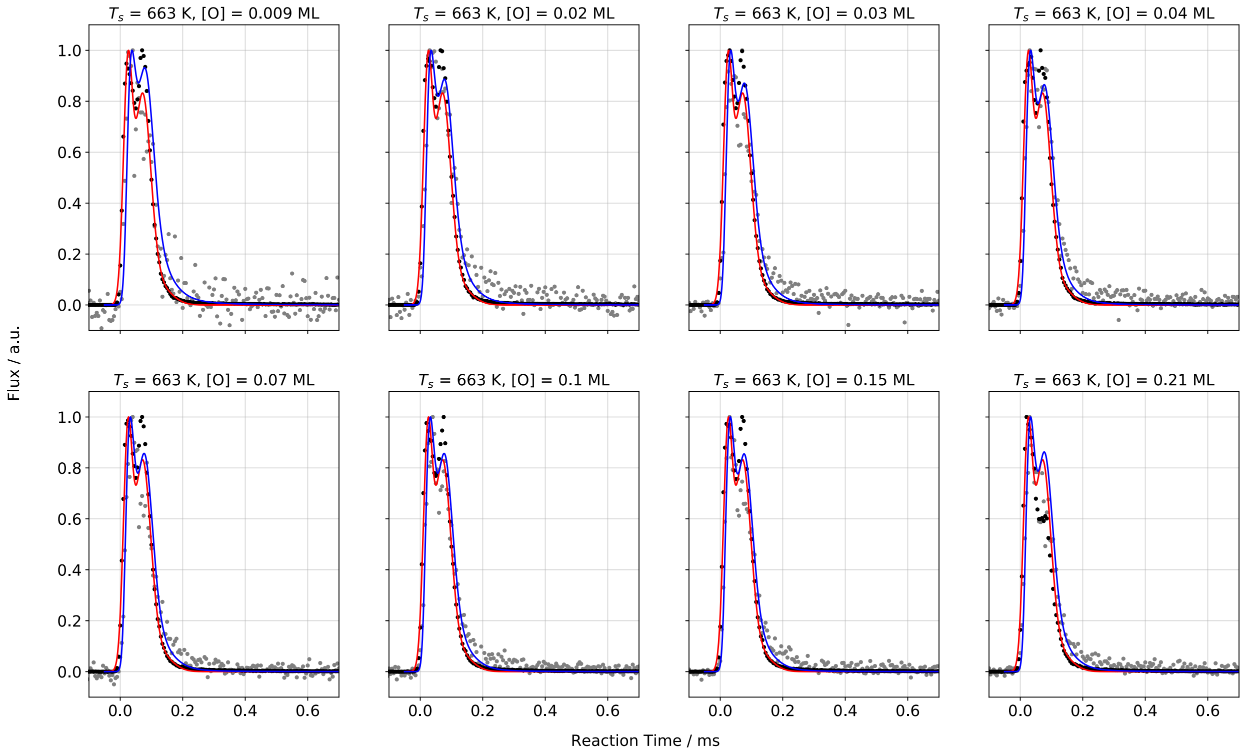

Figure S9: Same as Figure S7 but at 663 K.

S6. Oxidation Probability of NH3

Oxidation probabilities of $\mathrm{NH_3}$ were measured between 623 and $703\,\mathrm{K}$. If $I^0_{\mathrm{NH_3}}$ is the integral of the $\mathrm{NH_3}$ kinetic trace obtained for a clean surface ($[\mathrm{O}] = 0\,\mathrm{ML}$), and $I^\theta_{\mathrm{NH_3}}$ is the integral of the $\mathrm{NH_3}$ kinetic trace obtained for a mean oxygen coverage $[\mathrm{O}] = \theta$, then the oxidation probability is given by Eq. S29.

$$P_{\mathrm{ox}} = \frac{I^0_{\mathrm{NH_3}} - I^\theta_{\mathrm{NH_3}}}{I^0_{\mathrm{NH_3}}} \qquad \text{Eq. S29}$$

In practice, this depletion of the $\mathrm{NH_3}$ signal is only detectable for large oxygen coverages. The integrated $\mathrm{H_2O}$ signal, $I^\theta_{H_2O}$, is a more sensitive probe to obtain the reaction probability at lower coverages,

$$P_{\mathrm{ox}} = a \times (I^\theta_{\mathrm{H_2O}} - I^0_{\mathrm{H_2O}}) \qquad \text{Eq. S30}$$

where the scaling factor a is fixed so that the reaction probability at maximum coverage matches the result obtained from the $\mathrm{NH_3}$ loss data. This procedure is illustrated in Figure S10a) and b). Figure S10c) shows the derived oxidation probabilities as a function of oxygen coverage.

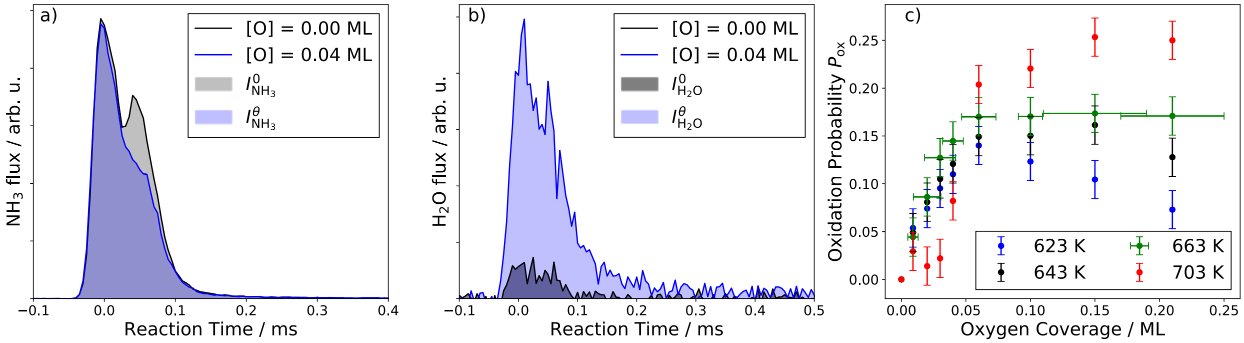

Figure S10: Ammonia Oxidation Probability as a function of Oxygen coverage and Temperature. *Panel (a) demonstrates the calculation of the reaction probability from the kinetic trace of NH_3 at the highest oxygen coverage considered. Panel (b) shows how this is used with the H_2O kinetic traces at the same coverage to obtain a scaling factor, used to calculate the probabilities at lower coverages using only the H_2O data. Panel (c) shows all calculated reaction probabilities at the four temperatures indicated in the legend.*

S7. TST Modelling of the Elementary Steps in NH_3 Oxidation

TST-DFT rate constants were computed for all steps in $\mathrm{NH_x}$ dehydrogenation ($x = 3, 2, 1$) using Eq. S31 and for $\mathrm{N^*O}$ formation using Eq. S32[7].

$$k_{\mathrm{TST}}(T) = \alpha_u \frac{k_b T}{h} \frac{N_{TS}}{N_{R_1} \times N_{R_2}} \frac{Q^{\ddagger}}{Q_{IS}} \exp\left(-\frac{E_0}{k_b T}\right) \quad \text{Eq. S31}$$

$$k_{\mathrm{TST}}(T) = \alpha_u \frac{k_b T}{h} \frac{Q^{\ddagger}}{Q_{\mathrm{O^*_{us}}} \times Q_{\mathrm{N^*}}} \exp\left(-\frac{E_0}{k_b T}\right) \quad \text{Eq. S32}$$

$$Q_{\mathrm{O^*_{us}/N^*}} = \sum_{i=1}^{n} g_i \prod_{j=1}^{m} \left(1 - \exp\left(-\frac{h\nu_j}{k_b T}\right)\right)^{-1} \exp\left(-\frac{E_i}{k_b T}\right) \quad \text{Eq. S33}$$

Here, α_u is the size of the unit cell ($149.58\ \text{Å}^2$); N_{TS}, N_{R_1} and N_{R_2} denote the degeneracy of the transition state and reactant R_1 and R_2 in their initial states. E_0 is the zero-point energy corrected reaction barrier height, $Q^{\ddagger}$ and Q_{IS} are the patition functions of the transition and initial state, respectively, within the harmonic oscillator approximation. As we have pointed out in the main text, our theoretical and experimental evidence indicate that the $\mathrm{N^*O}$ formation is the rate-limiting reaction which is why a more sophisticated approach to the partition function calculations is chosen here. The partition function $Q_{\mathrm{O^*_{us}/N^*}}$ of the initial states of nitrogen and oxygen are a sum over g_i degenerate sites within the unit cell and n is the number of different stable sites orthogonal to the mono-atomic step on Pd(332) which are weighted by the term $\exp\left(-\frac{E_i}{k_b T}\right)$ (see Figure S14). In the usual way, ν_j is the j-th harmonic frequency and E_i is the energy difference of the different stable sites to the global minimum. This formalism allows for calculation of a more sophisticated surface entropy within the harmonic oscillator approximation by taking the different adsorption sites into account.[8] The harmonic frequencies used for the partition functions are presented in Tables S1-S3. Structures and barrier heights are shown in Figures S12 and S13.

The TST-predicted rate constants are shown in Fig. S11, where one can clearly see that the smallest predicted rate constants belongs to the $N^{*}O$ formation reaction.

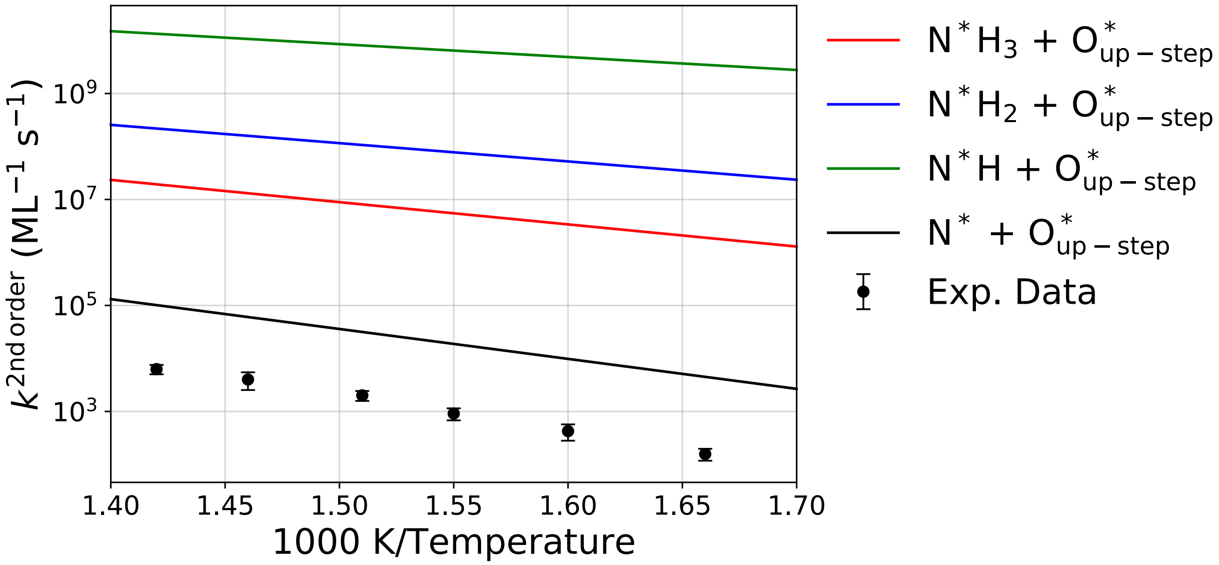

Figure S11: The TST-DFT predicted rate constant for each elementary steps towards $N^{*}O$ formation and comparison to experimentally derived rate constants.

The harmonic frequencies of NO^{*} at the up-step (Figure S16a) and on the terrace (Figure S16c) were also computed and are listed in Table S4.

.

Table S1: Harmonic frequencies of the initial state (IS) and transition state (TS) for each dehydrogenation reaction calculated with DFT using the RPBE functional. *All values are given in cm^{-1}. The structures are shown in **Figure S12**. All TSs have one imaginary frequency. The last row reports the degeneracy factor of the reactants (IS) and of the transitions state (TS) within the unit cell. Note that for reactants the degeneracy is the product of the site-degeneracy for oxygen atoms at the up-step multiplied by the site degeneracy of the NH_x reactant.*

Reaction	$\mathrm{N^*H_3 + O^*_{us}}$		$\mathrm{N^*H_2 + O^*_{us}}$		$\mathrm{N^*H + O^*_{us}}$	
vib. mode	IS	TS	IS	TS	IS	TS
1	3514.0	3571.5	3484.2	3436.9	3365.0	1995.1
2	3497.7	3496.7	3384.4	3037.8	734.6	1092.7
3	3378.0	3379.1	1469.9	1423.7	729.3	818.2
4	1594.6	1440.7	687.4	866.0	468.4	500.8
5	1587.0	735.0	631.4	701.8	443.6	496.8
6	1097.4	686.1	588.5	679.6	433.7	450.7
7	558.8	684.2	416.0	460.1	417.0	360.8
8	552.9	450.1	415.7	456.8	375.0	357.6
9	414.2	362.4	378.6	406.0	331.0	$293.2i$
10	374.6	329.0	329.3	338.8	−	−
11	316.4	288.4	309.2	251.6	−	−
12	287.0	226.6	161.2	$106.2i$	−	−
13	120.5	101.8	−	−	−	−
14	114.5	89.8	−	−	−	−
15	9.2	$227.0i$	−	−	−	−
degeneracy	4×4	7	4×4	4	4×4	4

Table S2: Harmonic frequencies of N^* and O^* reactants as well as for the transition state (TS) of the N^*O reaction calculated with DFT using the RPBE functional. *All values are given in cm^{-1}. Frequencies were computed for N^* at the up-step (see **Figure S14**f) and at fcc terrace sites (see **Figure S14**g-j) as well as O^* at the up-step (see **Figure S14**a) and at fcc terrace sites (see **Figure S14**a-e).*

Configuration	1	2	3	4	5	6
	Nitrogen			Oxygen		
up-step	518.6	509.5	474.1	423.6	359.5	335.8
fcc-terrace	513.0	505.8	472.9	409.5	360.1	337.0
TS	504.6	463.0	422.9	330.5	300.6	$362.4i$

Table S3: Same as Table S2 but using the PBE functional

Configuration	1	2	3	4	5	6
	Nitrogen			Oxygen		
up-step	531.0	521.5	470.2	438.1	372.9	354.7
fcc-terrace	523.8	514.6	479.3	422.0	378.1	372.5
TS	516.5	466.6	438.9	341.5	313.1	$346.5i$

Table S4: Listed are the harmonic frequencies of NO at the different adsorption sites using PBE and RPBE. *All values are given in cm^{-1}.*

PBE	1	2	3	4	5	6
NO (Figure S16a)	1531.9	450.9	433.6	303.0	154.4	136.0
NO (Figure S16c)	1549.8	442.0	440.6	309.0	156.8	152.9
RPBE	1	2	3	4	5	6
NO (Figure S16a)	1509.2	443.5	424.4	292.3	150.7	132.0
NO (Figure S16c)	1526.5	433.7	431.3	296.9	151.6	149.6

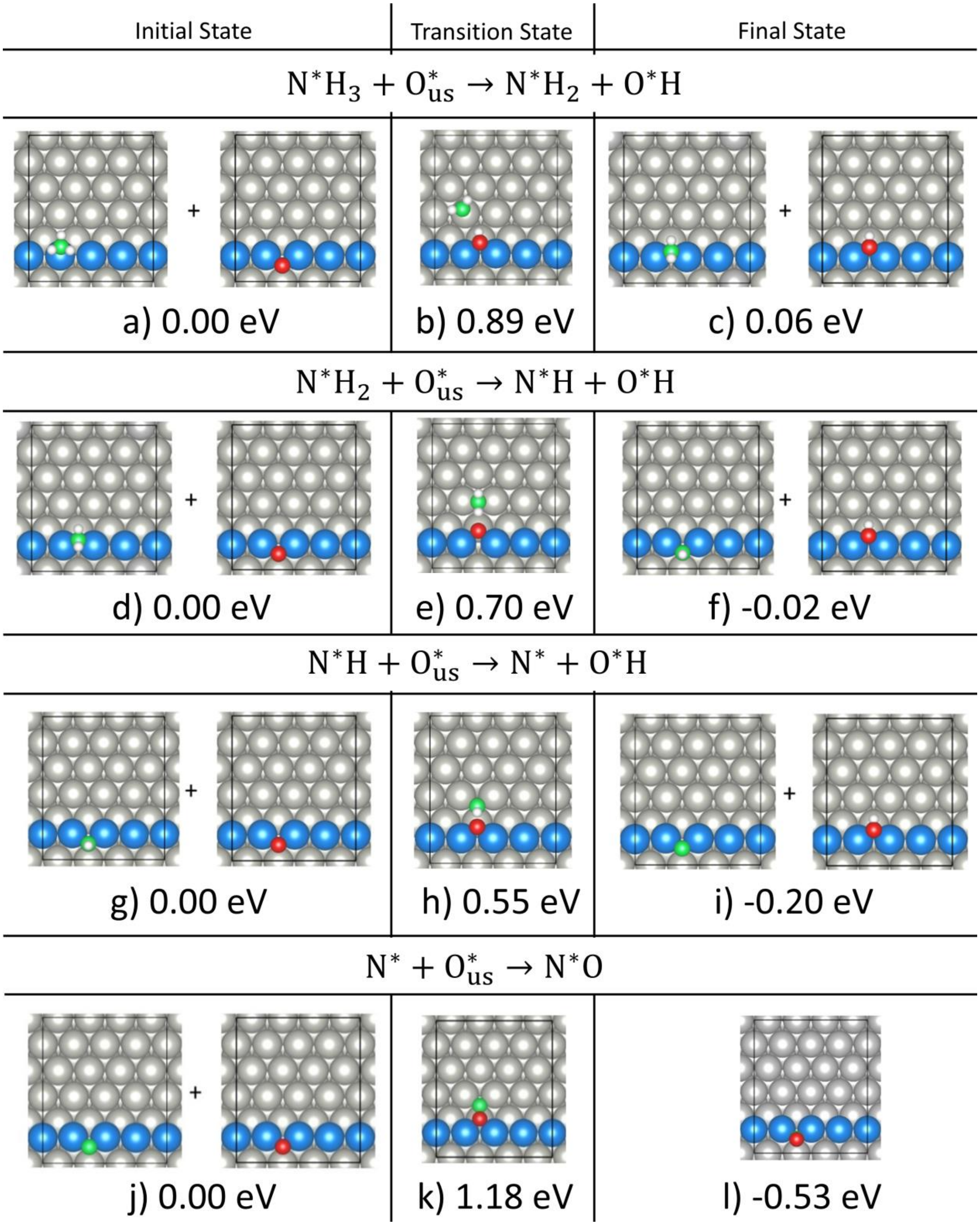

Figure S12: Calculated structures for the reaction of N^*H_3, N^*H_2, N^*H and N^* with oxygen bound to the up-step site. *RPBE energies are presented without zero-point correction. The initial and final state structures are the most stable geometry and do not represent the images used in the nudge-elastic band calculations. Palladium, oxygen, hydrogen and nitrogen atoms are shown in gray, red, white and green, respectively. The monoatomic step is shown in blue.*

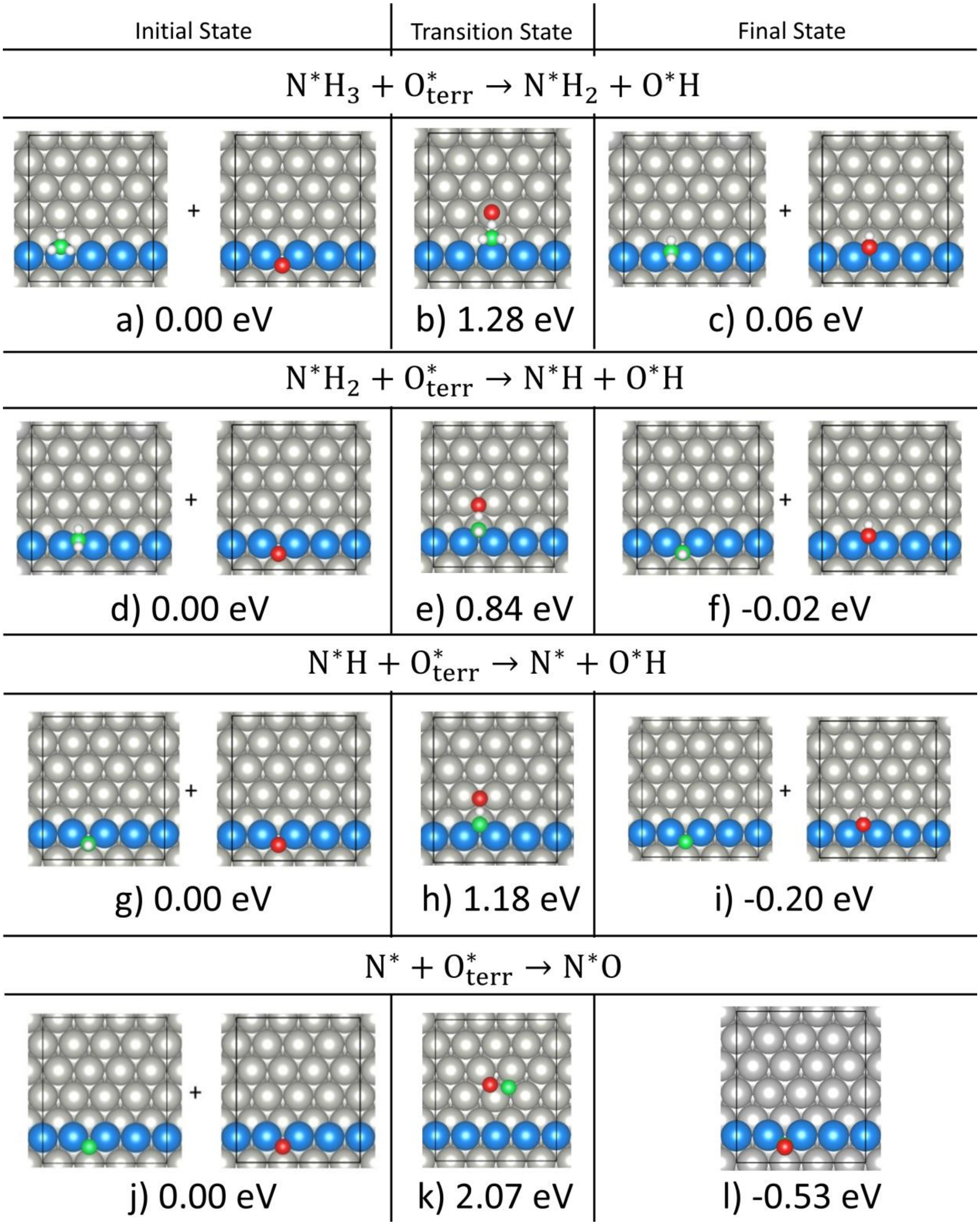

Figure S13: Calculated structures for the reaction of N^*H_3, N^*H_2, N^*H and N^* with oxygen bound to the terrace site. *RPBE energies are presented without zero-point correction. The initial and final state structures are the most stable geometry and do not represent the images used in the nudge-elastic band calculations. Palladium, oxygen, hydrogen and nitrogen atoms are shown in gray, red, white and green, respectively. The monoatomic step is shown in blue.*

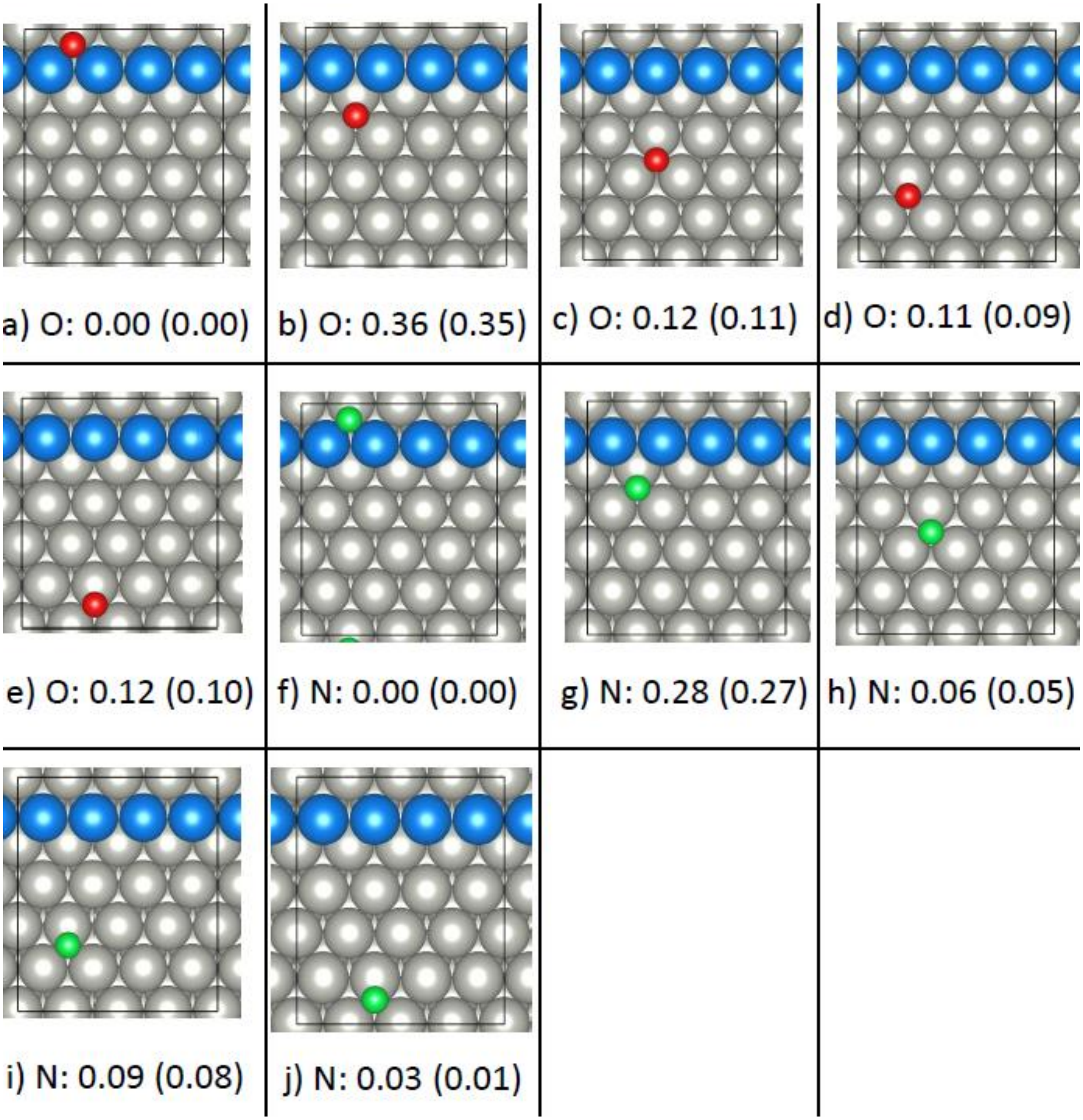

Figure S14: Energies with respect to the global minimum for the O^* (red) and N^*(green) at various binding sites on Pd(332). *Each site has a fourfold degeneracy in the unit cell. All values are calculated using PBE (RPBE) and are given in eV. Palladium gray, except for the step atoms, which are shown in blue. The black solid line indicates the 4x1 periodic unit cell used for all calculations.*

S8. Influence of Oxygen Coverage on NO Desorption

a. Experimental desorption rate constants

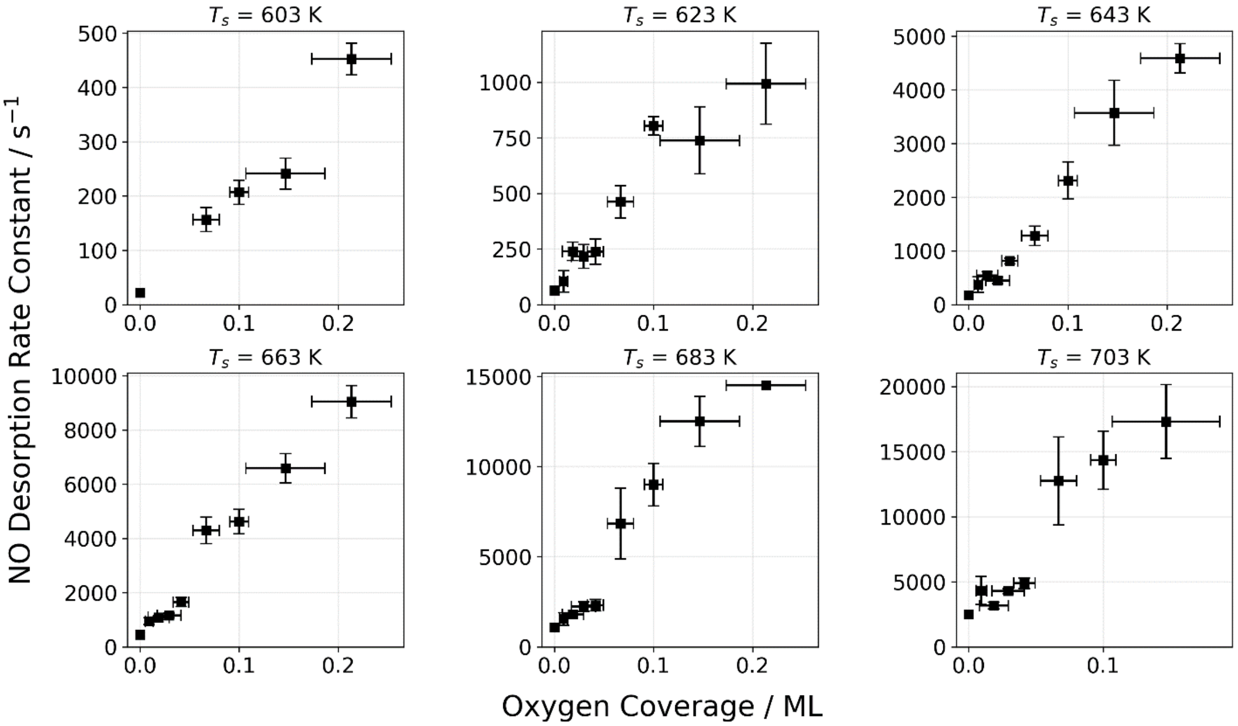

Figure S15: The experimentally derived desorption rate constants of NO from Pd(332) as a function of the steady-state oxygen coverage and temperature. *The zero-coverage value is taken from our previous work.[9]*

In Figure S15, we show the experimentally derived desorption rate constants of NO as function of the steady-state oxygen coverage for six surface temperatures ranging from 603 K to 703 K. The desorption rate constants increase with coverage for all cases. Table S5 shows the same results as Arrhenius parameters. The Arrhenius pre-factor is seen to be nearly independent of O-coverage, whereas the Arrhenius activation energy drops by about 0.1 eV with respect to the desorption from the oxygen free Pd(332) surface.[9] These findings are consistent with an independent experiment where we measured the kinetics of NO trapping-desorption for clean and oxygen atom coverage saturated Pd(332) surface—see main text.

Table S5: Oxygen coverage dependent Arrhenius parameters for NO desorption from Pd (332).

	Steady-state oxygen coverage / ML							
	0.009(4)	0.02(1)	0.03(1)	0.041(8)	0.07(1)	0.100(9)	0.15(4)	0.21(4)
A_d/s^{-1}	$10^{15.7}$	$10^{15.7}$	$10^{15.7}$	$10^{15.7}$	$10^{16.0}$	$10^{16.0}$	$10^{15.9}$	$10^{16.0}$
E_a/ eV	1.68	1.67	1.67	1.66	1.64	1.63	1.60	1.60

b. DFT calculations

Figure S16 shows relative energies of N^*O at different surface sites as obtained with DFT using PBE and RPBE functionals without zero-point energy correction. The energy-zero corresponds to the structure with the most stable binding (Figure S16a).

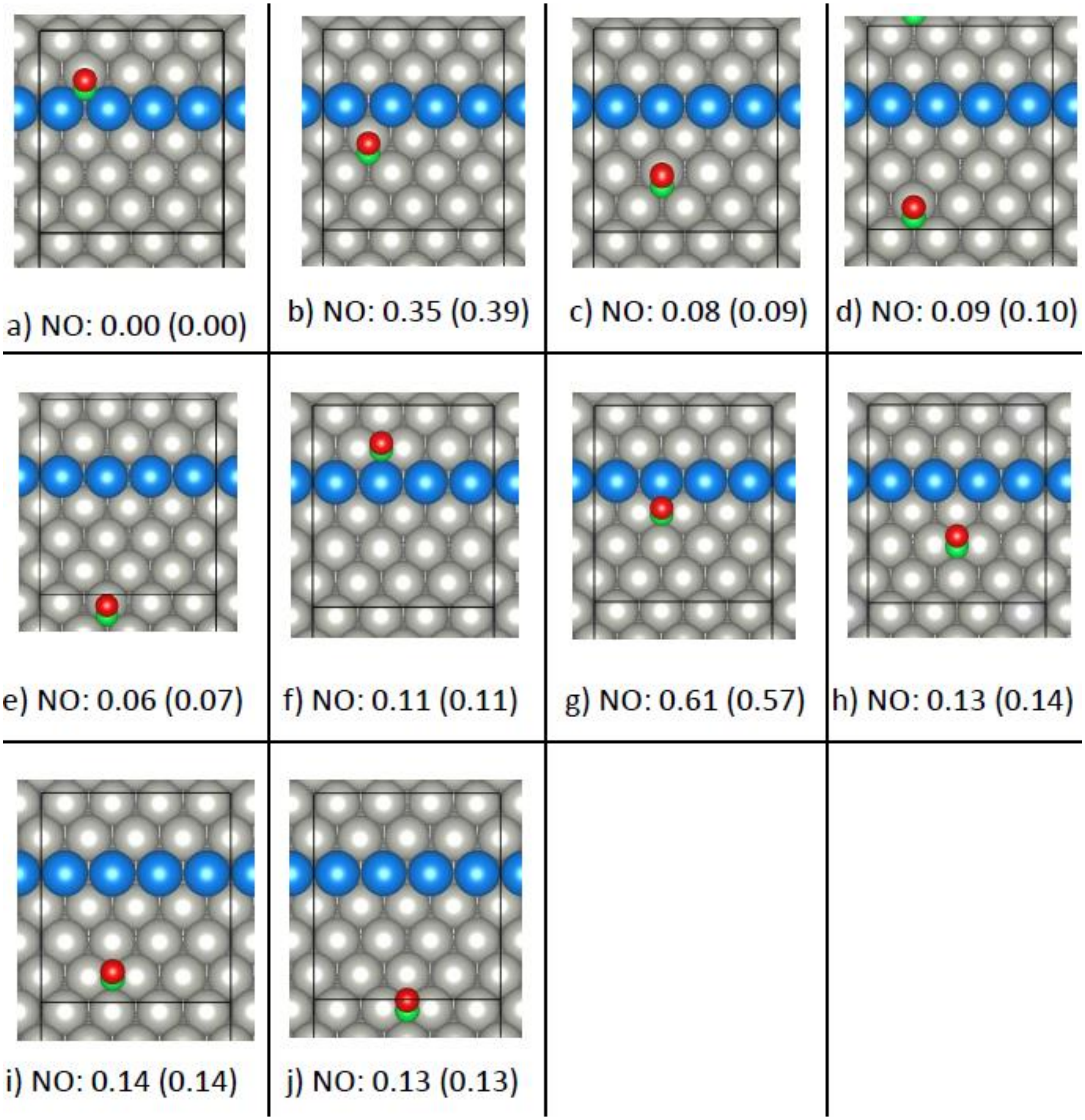

Figure S16: The relative energies of N^*O adsorbed at various binding sites of Pd(332) using PBE (RPBE). *The values are given in eV. Palladium, oxygen and nitrogen atoms are shown in gray, red and green, respectively. The monoatomic step is shown in blue. The black solid line indicates the 4x1 periodic unit cell used for all calculations.*

We next consider the interaction energies of N^*O and O^*, which are calculated from Eq. S34.

$$E_{int} = E_{i=1,2} - (E_{i=1,2}^{NO} + E^{O}) + E_{\mathrm{Pd(332)}} \qquad \text{Eq. S34}$$

Here, E_{int} denotes the change in energy of N^*O due to the presence of n O^* atoms at nearby binding sites, where in our calculations $n = 1 - 4$. E_i denotes the DFT-computed energy for a given configuration of N^*O with n O^* atoms. Two values of i are considered, one denoting N^*O bound at the up-step site (Fig. S16a) and the other denoting N^*O bound at the *fcc* hollow of the terrace (Fig. S16d). E_i^{NO} denotes the DFT computed energy for NO at the ith binding site in the absence of O^* adsorbates. E^{O} denotes the energy of the system for a given configuration of n O^* atoms in the absence of N^*O. In Eq. S34, a difference is taken between three DFT energies – each with the energy of the Pd atoms included. This requires us to add the energy of the Pd(332) unit cell, denoted $E_{\mathrm{Pd(332)}}$, to balance the equation.

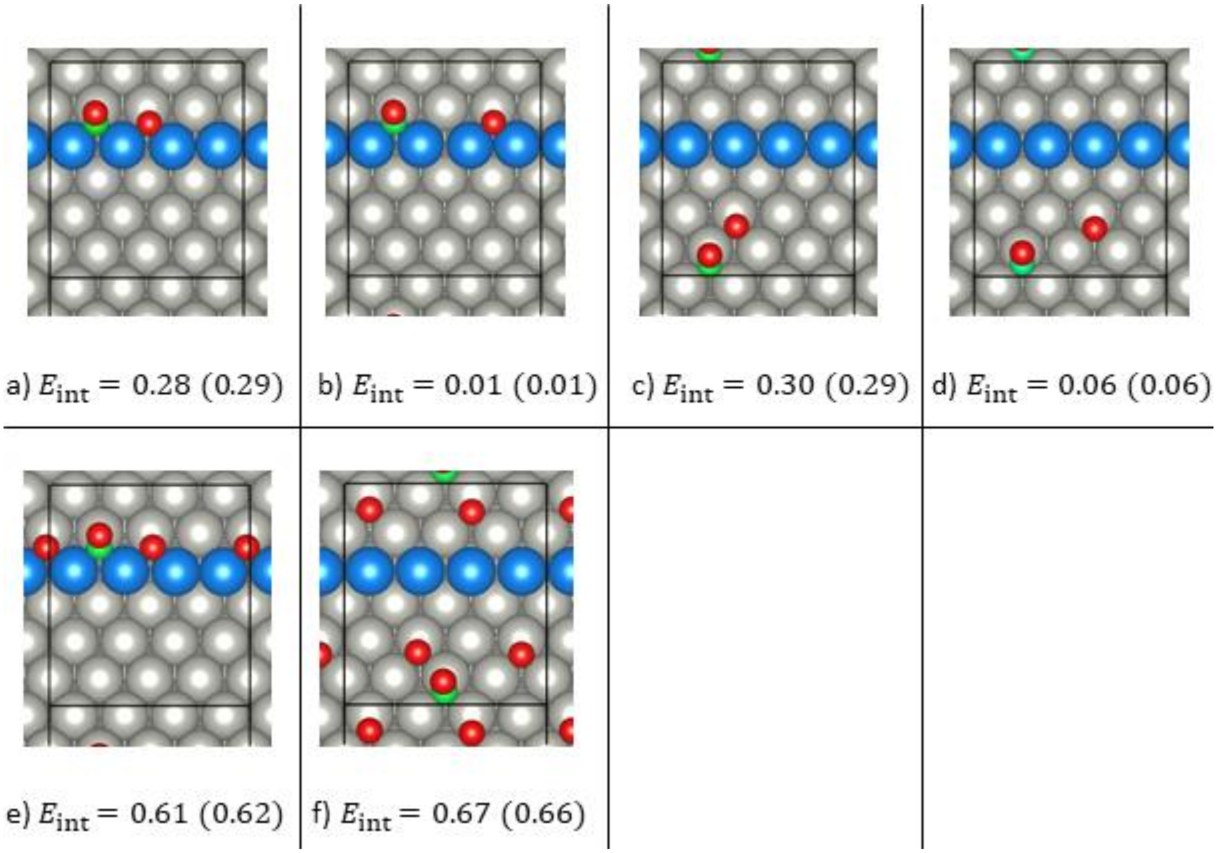

Figure S17: Interaction energies of NO^* with O^*. *Energies are shown for different configurations on Pd(332). Values are computed using PBE (RPBE) and are given in eV. Palladium, oxygen and nitrogen atoms are shown in gray, red and green, respectively. The monoatomic step is shown in blue.*

References

1. Chadwick, H.; Guo, H.; Gutiérrez-González, A.; Menzel, J. P.; Jackson, B.; Beck, R. D., Methane dissociation on the steps and terraces of Pt(211) resolved by quantum state and impact site. *J. Chem. Phys.* **2018,** *148* (1).
2. HiPace 700 Turbopump Operating Instructions. In *Technical data and dimensions*, Pfeiffer Vacuum: Germany, 2016.
3. Stabil-Ion Vacuum Measurement Controller. In *Gauge Scale Factor*, MKS Instruments: USA, 2020.
4. Shirhatti, P. R.; Geweke, J.; Steinsiek, C.; Bartels, C.; Rahinov, I.; Auerbach, D. J.; Wodtke, A. M., Activated Dissociation of HCl on Au(111). *J. Phys. Chem. Lett.* **2016,** *7* (7), 1346-1350.
5. Schwarzer, M.; Borodin, D.; Wang, Y.; Fingerhut, J.; Kitsopoulos, T. N.; Auerbach, D. J.; Guo, H.; Wodtke, A. M., Cooperative adsorbate binding catalyzes high-temperature hydrogen oxidation on palladium. *Science* **2024,** *386* (6721), 511-516.
6. Borodin, D.; Golibrzuch, K.; Schwarzer, M.; Fingerhut, J.; Skoulatakis, G.; Schwarzer, D.; Seelemann, T.; Kitsopoulos, T.; Wodtke, A. M., Measuring Transient Reaction Rates from Nonstationary Catalysts. *Acs. Catal.* **2020,** *10* (23), 14056-14066.
7. Kramer, Z. C.; Gu, X. K.; Zhou, D. D. Y.; Li, W. X.; Skodje, R. T., Following Molecules through reactive networks: Surface catalyzed decomposition of methanol on Pd(111), Pt(111), and Ni(111). *J. Phys. Chem. C* **2014,** *118* (23), 12364-12383.
8. Blöndal, K.; Sargsyan, K.; Bross, D. H.; Ruscic, B.; Goldsmith, C. F., Configuration Space Integration for adsorbate partition functions: The effect of anharmonicity on the thermophysical properties of CO-Pt(111) and CH3OH-Cu(111). *ACS Catal.* **2023,** *13* (1), 19-32.
9. Borodin, D.; Rahinov, I.; Fingerhut, J.; Schwarzer, M.; Hörandl, S.; Skoulatakis, G.; Schwarzer, D.; Kitsopoulos, T. N.; Wodtke, A. M., NO binding energies to and diffusion barrier on Pd obtained with velocity-resolved kinetics. *J. Phys. Chem. C* **2021,** *125* (21), 11773-11781.
